# Functional analysis of *StWRKY75* gene in response to heat stress in potato (*Solanum tuberosum* L.)

**DOI:** 10.3389/fpls.2025.1617625

**Published:** 2025-06-30

**Authors:** Xi Zhu, Xiaoqin Duan, Yasir Majeed, Junfu Luo, Nengkang Guan, Haifei Zheng, Huafen Zou, Hui Jin, Zhuo Chen, Yu Zhang

**Affiliations:** ^1^ Key Laboratory of Tropical Fruit Biology, Ministry of Agriculture and Rural Affairs, South Subtropical Crops Research Institute, Chinese Academy of Tropical Agricultural Sciences, Zhanjiang, Guangdong, China; ^2^ Key Laboratory of Hainan Province for Postharvest Physiology and Technology of Tropical Horticultural Products, South Subtropical Crops Research Institute, Chinese Academy of Tropical Agricultural Sciences, Zhanjiang, Guangdong, China; ^3^ National Key Laboratory for Tropical Crop Breeding, Sanya Research Institute, Chinese Academy of Tropical Agricultural Sciences, Sanya, China; ^4^ State Key Laboratory of Aridland Crop Science, Gansu Agricultural University, Lanzhou, China; ^5^ College of Agronomy, Gansu Agricultural University, Lanzhou, China; ^6^ College of Tropical Crops, Yunnan Agricultural University, Pu’er, China

**Keywords:** *StWRKY75*, *Solanum tuberosum*, heat stress, transgenic, photosynthetic efficiency

## Abstract

Heat stress severely restricts potato tuber growth and development, yet the roles of WRKY transcription factors (TFs) in mediating heat responses remain poorly understood. Using quantitative reverse transcription PCR (qRT-PCR), we identified *StWRKY75* in potato cultivars ‘*Atlantic*’ and ‘*Desiree*’ as a heat-inducible gene, showing significant upregulation under 30°C and 35°C treatments. Phylogenetic analysis classified *StWRKY75* into the WRKY Group II family, with close evolutionary homology to tomato *SlWRKY75*, and subcellular localization confirmed its nuclear targeting. To characterize its function, we generated transgenic lines overexpressing *StWRKY75* (OE) or knockdown by RNA interference (RNAi). Under heat stress conditions, OE plants demonstrated superior thermotolerance compared to non-transgenic (NT) controls, manifested by improved growth parameters (plant height, tuber weight per plant, fresh weight, dry weight, root fresh weight, and root dry weight), enhanced activities of antioxidant enzymes (ascorbate peroxidase [APX], catalase [CAT], peroxidase [POD], and superoxide dismutase [SOD]), increased proline accumulation, elevated photosynthetic efficiency, and reduced malondialdehyde (MDA) content. Conversely, RNAi lines exhibited compromised thermotolerance with reversed growth parameters and biochemical characteristics. qRT-PCR analysis revealed that *StWRKY75* positively regulated key heat-stress responsive genes, including those encoding antioxidant enzymes (*StAPX, StCAT, StSOD, StPOD*), proline biosynthesis Pyrroline-5-carboxylate synthase (*StP5CS*), and heat shock proteins (*StHSP20, StHSP70, StHSP90*). These findings demonstrate that *StWRKY75* acts as a positive regulator of potato thermotolerance by enhancing growth, antioxidant capacity, proline metabolism, photosynthesis, and heat-responsive gene expression, providing a valuable target for improving crop heat resilience in breeding programs.

## 1 Introduction

Potatoes (*Solanum tuberosum* L.), a critical component of global food systems, serve as a multifaceted solution to address food security challenges exacerbated by climate change and population growth (Devaux et al., 2020). As the fourth most widely cultivated crop worldwide, following rice, wheat, and maize, potatoes offer exceptional nutritional value, high yield potential, and adaptability across diverse agro-ecologies (Adekanmbi et al., 2024). Hailed as a “climate-smart” staple, their resilience to marginal soils and suboptimal climates positions them as a strategic choice for enhancing global food security and nutrition (Kumar et al., 2021). However, exposure to temperatures exceeding 30°C detrimentally impacts potato growth and yield, causing tuber discoloration, impaired starch accumulation, disrupted dormancy, and premature sprouting, consequences directly linked to global warming induced by climate change (Rykaczewska, 2015). As temperate crops, potatoes thrive optimally at ≤25°C; while transient exposure to 25–30°C is tolerated, productivity declines significantly under such conditions (Singh et al., 2020). Prolonged heat stress (>30°C) induces stomatal closure for water conservation, halving plant development rates (Aien et al., 2017). Extensive research identifies temperature as the primary determinant of potato productivity, with elevated heat emerging as a dominant stressor compared to other environmental variables (Bender and Sentelhas, 2020; Gonzalez-Jimenez et al., 2023; Zhu et al., 2024; Guillemette et al., 2025).

Plants have evolved sophisticated mechanisms to combat abiotic stresses, with TFs playing a central regulatory role. The WRKY TF superfamily, unique to algae and higher plants, is pivotal in mediating responses to biotic and abiotic stresses (Pandey and Somssich, 2009). Structurally, WRKY proteins are defined by an N-terminal DNA-binding domain containing a conserved heptapeptide (e.g., WRKYGQK, with variants such as WRKYGKK and WRKYGMK) and a C-terminal zinc-finger motif, primarily classified as C_2_H_2_ or C_2_HC types, though atypical structures like CX_29_HXH also exist (Eulgem et al., 2000; Phukan et al., 2016). Grouped into three categories (I, II, or III) based on the number of DNA-binding domains and zinc-finger arrangements, their modular architecture enhances regulatory functions in stress responses and plant development (Wani et al., 2021). WRKY TFs modulate gene expression through dual mechanisms: direct binding to W-box cis-elements in target promoters to activate or repress transcription, and forming protein complexes with co-factors to enhance transcriptional activity (Eulgem et al., 2000; Phukan et al., 2016). Notably, many WRKY promoters contain W-box elements, enabling autoregulation and cross-regulatory networks via homo/hetero dimerization with other WRKYs, collectively shaping plant transcriptional processes (Ciolkowski et al., 2008). Since the first WRKY gene (SPF1 in sweet potato) was identified in 1994 (Ishiguro and Nakamura, 1994), this family has been extensively studied in various plants under abiotic stresses. For example, in *Arabidopsis*, *AtWRKY25/26/3*3 regulate thermotolerance (Li et al., 2011), while *AtWRKY6/26/30/40/45/75* are upregulated under reactive oxygen species (ROS) treatment (Cheong et al., 2002). Transgenic rice seedlings overexpressing *OsWRKY11* exhibit enhanced tolerance to both drought and heat stress (Wu et al., 2009), and *OsWRKY72* mediates salt tolerance (Song et al., 2010). The tomato *SlWRKY3* contributes to salt stress regulation (Hichri et al., 2017), while apple *MdWRKY30* enhances resistance to salt and osmotic stress in transgenic calli through the modulation of stress-responsive genes (Dong et al., 2020). In maize, *ZmWRKY106* confers heat tolerance, whereas in wheat, *TaWRKY30* enhances drought resistance (Wang et al., 2018; El-Esawi et al., 2019). These studies highlight WRKY TFs as key players in maintaining plant growth under stress, often by enhancing antioxidant enzyme activities, proline accumulation, and ROS scavenging gene expression (Duan et al., 2023; Yu et al., 2023; Wang et al., 2024). The WRKY transcription factor (TF) family plays a crucial role in plant stress responses, including heat tolerance. In potato (Solanum tuberosum), 79 StWRKY genes have been identified through genome-wide analysis (Zhang et al., 2017), with sequences available in the Potato Genome Sequencing Consortium (PGSC) database (http://solanaceae.plantbiology.msu.edu/pgsc_download.shtml). However, the functional characterization of *StWRKY75* in heat stress responses remains unexplored. This qRT-PCR analysis provided the evidence that *StWRKY75* confers heat tolerance in potato, which is validated by the up-regulated expression of *StWRKY75* genes in two cultivars of potatoes (*Atlantic* and *Desiree*) under varied heat stress treatments at various time intervals. This upregulated expression profile of the *StWRKY75* gene provides a strong base for further analyzing the functional validation of this gene under high-temperature stress conditions. Furthermore, extensive evidence identifies Desiree as a potato cultivar with high sensitivity to both drought and elevated temperature (Basu and Minhas, 1991; Stark et al., 2013; Rolando et al., 2015). At the same time, Atlantic exhibits pronounced thermosensitivity, demonstrating significant reductions in both photosynthetic efficiency and tuber yield under evaluated heat stress conditions. This highlights Atlantic as a heat-sensitive cultivar, with its physiological and agronomic performance being markedly compromised under elevated temperatures (Ahn et al., 2004; Gautam et al., 2021; 2024). Given their established susceptibility to elevated temperatures, the potato cultivars *Desiree* and *Atlantic* were chosen as model genotypes in this study to assess heat stress-induced alterations in growth, physiological, and photosynthetic performance.

This study aims to elucidate the function of *StWRKY75* in potato (cultivars ‘*Atlantic*’ and *‘Desiree’*) thermotolerance by integrating multiple-sequence and phylogenetic analysis, expression profiling, subcellular localization, and transgenic validation. Specifically, we examine how *StWRKY75* modulates growth-related traits, antioxidant enzyme activities, proline accumulation, MDA contents, photosynthetic efficiency, and the expression of heat-responsive genes (*StAPX*, *StSOD*, *StPOD*, *StCAT*, *StP5CS, StHSP20, StHSP70*, and *StHSP90*) to enhance stress tolerance. Our findings provide mechanistic insights into *StWRKY75*-mediated heat resistance, offering potential targets for improving potato resilience to climate change.

## 2 Materials and methods

### 2.1 Plant material and experimental design

The apical buds of tissue-cultured seedlings from two potato cultivars with inconsistent genetic backgrounds for heat stress, ‘*Atlantic*’ (heat-sensitive) and ‘*Desiree*’ (heat-tolerant), were transferred to MS medium and cultured for 28 days under controlled conditions (16-hour light/8-hour dark photoperiod, 2800 lux light intensity, 20°C). The seedlings were then transplanted into autoclaved substrate soil and cultivated in a growth chamber for 14 days (16-hour light/8-hour dark photoperiod, 2800 lux light intensity, 20°C, 75% relative humidity). Uniform plants were selected and transplanted into pots (26 cm × 27 cm × 18 cm) containing a soil-vermiculite mixture (1:1, v/v) and grown for five weeks under soil moisture maintained at 70–75%. To investigate the response of the *StWRKY75* gene to drought, heat, and salt stress, experimental conditions included drought stress induced by 10% and 20% polyethylene glycol 6000 (PEG6000), heat stress at 30°C and 35°C, and salt stress with 75 mM and 150 mM sodium chloride (NaCl). Plants were exposed to these abiotic stresses for 0, 1, 2, 4, 8, 16, 24, and 48 hours, followed by leaf sampling for mRNA expression analysis. To assess *StWRKY75* expression levels, 432 potted plants were analyzed, encompassing two potato cultivars, two drought stress treatments (10% and 20% PEG6000), two heat stress treatments (30°C and 35°C), two salt stress treatments (75 mM and 150 mM NaCl), eight time points (0–48 hours), three biological replicates, and three technical replicates (pots). For quantitative evaluation of physiological parameters, photosynthetic indices, and heat-responsive genes under heat stress, 14-day-old transgenic and NT lines of both cultivars from growth chambers were transplanted into pots (26 cm × 27 cm × 18 cm) containing a soil-vermiculite mixture (1:1, v/v), grown for five weeks under 70–75% soil moisture, and subjected to heat stress (30°C and 35°C) for 48 hours. A total of 378 seedlings were evaluated, including two cultivars, seven genotypes per cultivar (one NT line, three *StWRKY75* overexpression lines, and three RNAi-mediated silencing lines), two heat treatments (30°C and 35°C), three biological replicates, and three technical replicates.

### 2.2 Phylogenetic and multiple-sequence alignment analysis

Multiple-sequence alignment of StWRKY75 with orthologs from 18 additional plant species was conducted using CLUSTALW (version 1.83) (Thompson et al., 1994). Conserved protein motifs were subsequently identified through the same alignment analysis. Phylogenetic reconstruction was performed using MEGA X (version 4.1) (Dereeper et al., 2010), employing the Neighbor-Joining (NJ) method with 1000 bootstrap replicates to assess nodal support.

### 2.3 RNA extraction and quantitative PCR analysis

Total RNA was extracted from the samples using the TRIzol reagent (Invitrogen, Carlsbad, CA, USA) according to the manufacturer’s protocol. First-strand cDNA was synthesized from 1 µg of total RNA using the TransScript First-Strand cDNA Synthesis Kit (TransGen Biotech, Beijing, China). The qRT-PCR was performed on a LightCycler 480 II instrument (Roche, Rotkreuz, Switzerland) in 20 µL reaction mixtures. Each reaction contained 100 ng of cDNA, 0.8 µL of gene-specific primers (see **Supplementary Table S1**), and 10 µL of SYBR Premix Ex Taq (2×) (Takara, Tokyo, Japan). The amplification conditions were as follows: initial denaturation at 94°C for 2 min, followed by 34 cycles of denaturation at 94°C for 30 s, primer annealing at 60°C for 34 s, and extension at 72°C for 30 s. The *Stef1α* gene was used as an internal control to normalize gene expression levels. Relative gene expression was quantified using the 2^−ΔΔCt^ method (Livak and Schmittgen, 2001) based on cycle threshold (CT) values. To ensure reproducibility, three biological replicates were analyzed, each with three technical replicates. The primer sequences used in this study are provided in **Supplementary Table S1**.

### 2.4 Plasmid construction and genetic transformation

The *StWRKY75* coding sequence (GenBank ID: NM_001288675.1) was PCR-amplified using specific primers (forward: 5’-CTCGACATGGAGAATTATGCAACAATAT-3’; reverse: 5’-GTCGACAAAAGGAAGTATAGATTTGCATCT-3’; Bioeditas, Shaanxi, China) and cloned into the pBI121-EGFP vector following established protocols (Li et al., 2020). RNAi lines were generated as previously described (Lu et al., 2019). The sense cDNA fragment was amplified using *Eco*R I/*Kpn* I*-*restriction sites primers and cloned into pHANNIBAL (pHAN-StWRKY75-R). Similarly, the antisense fragment was amplified with *Hind* III/*Xba* I primers and inserted to create pHAN-StWRKY75-RF. The pHAN-StWRKY75 construct was then sub-cloned into the pART vector at the *Xho* I and *Xba* I restriction sites, resulting in the pART-StWRKY75-RNAi construct. The construct was transformed into *Agrobacterium tumefaciens* LBA4404 and cultured (28°C, 48 h) for subsequent plant transformation. Transformants were verified by PCR amplification using RNAi-specific primers: 5’-TGGAGAATTATGCAACAATATTTC-3’ (forward) and 5’-CCATCTATAACCATCAAGAAT-3’ (reverse), NPT II selection marker primers: 5’-ATGACTGGGCACAACAGACAATCG-3’ (forward) and 5’-TCAGAAGAACTCGTCAAGAAGGCG-3’ (reverse). The transformed *Agrobacterium tumefaciens* culture was maintained in an LB medium supplemented with 50 mg/L each of spectinomycin and gentamicin at 28°C for 48 hours. Following centrifugation (5,000 × g, 10 min), bacterial cells were resuspended in MS liquid medium to achieve OD600 = 0.3. Surface-sterilized potato stem segments (2 cm) were cultured on an MS medium containing 7.4 g/L agar, 30 g/L sucrose, 0.5 mg/L 6-benzylaminopurine (6-BA), 2.0 mg/L zeatin (ZT), 0.2 mg/L gibberellic acid(GA3), 1.0 mg/L indole-3-acetic acid (IAA) (pH adjusted to 5.8). The explants were immersed in Agrobacterium suspension for 10 min, then co-cultivated in darkness (72 h, 25°C). Subsequently, samples were transferred to the selection medium (composition as above, supplemented with 300 mg/L timentin, 100 mg/L kanamycin), with media refreshed biweekly. The selection medium was replaced every two weeks to maintain antibiotic pressure. Developing shoots that exhibited stable antibiotic resistance were subsequently excised and transferred to a rooting medium consisting of MS basal salts, 7.4 g/L agar, 30 g/L sucrose, 300 mg/L timentin, and 100 mg/L kanamycin to promote adventitious root formation.

### 2.5 Subcellular localization of StWRKY75 in tobacco epidermal cells

The protein-coding sequence of StWRKY75 was amplified using primers (forward: 5′-ATGGAGAATTATGCAACAATAT-3′; reverse: 5′-AAAAGGAAGTATAGATTTGCATCT-3 ′) and cloned into the pCAM35s-GFP expression vector. The recombinant plasmid was introduced into *Agrobacterium tumefaciens* strain GV3101. Transient transformation of tobacco (*Nicotiana benthamiana*) epidermal cells was performed following the protocol described by Sparkes et al. (2006). GFP fluorescence was visualized after 48 hours of post-infiltration using a Leica TCS SP8 confocal laser scanning microscope (Leica Microsystems, Wetzlar, Germany).

### 2.6 Assessment of growth-related traits

Apical buds of transgenic and NT tissue-cultured seedlings from two uniform potato cultivars, ‘*Atlantic*’ and ‘*Desiree*’, were transferred to MS medium and cultured for 28 days under controlled conditions (16-hour light/8-hour dark photoperiod, 2800 lux light intensity, 20°C). The seedlings were then transplanted into autoclaved substrate soil and cultivated in a growth chamber for 14 days (16-hour light/8-hour dark photoperiod, 2800 lux light intensity, 20°C, 70% relative humidity). Uniformly growing plants were selected and transplanted into pots (26 cm × 27 cm × 18 cm) containing a soil-vermiculite mixture (1:1, v/v) and grown for five weeks under soil moisture maintained at 70–75%. Plants with consistent growth were subjected to heat stress (30°C and 35°C) for five weeks, followed by growth parameter analysis. Plant height was measured as the distance from the soil surface to the stem apex, while tuber yield per plant and biomass (fresh and dry weights of shoots and roots) were evaluated under different heat stress conditions. Fresh weights were recorded immediately after treatment, and dry weights were determined after drying samples to constant weight in a 70°C oven. A total of 378 seedlings were assessed, encompassing two cultivars, seven genotypes per cultivar (1 NT line, 3 *StWRKY75* overexpression lines, and 3 RNAi-mediated silencing lines), two heat treatments (30°C and 35°C), three biological replicates, and three technical replicates.

### 2.7 Assessment of physiological indicators

The key physiological parameters, including the activities of SOD (Giannopolitis and Ries, 1977), CAT (Aebi, 1984), POD (Maehly and Chance, 1954), and APX (Nakano and Asada, 1981), as well as the levels of proline (Bates et al., 1973) and MDA (Heath and Packer, 1968), were assayed using established protocols (**Supplementary Data 1**). The study evaluated 378 seedlings from two cultivars, each comprising seven genotypes (an NT control, three *StWRKY75*-overexpression lines, and three *StWRKY75*-RNAi lines), under two heat stress regimes (30°C and 35°C) in a completely randomized design.

### 2.8 Assessment of photosynthetic indicators

The photosynthetic capacity of plants was assessed by examining the third fully developed leaf from the shoot apex, with measurements conducted during mid-morning hours (9:30-11:30 AM). Gas exchange measurements, comprising net photosynthesis (Pn), transpiration (E), and stomatal conductance (Gs), were collected using Li-COR’s 6400XT portable photosynthesis monitoring equipment. Measurements were conducted under controlled environmental conditions: photosynthetic photon flux density (PPFD) of 1500 μmol·m^−2^·s^−1^, atmospheric CO_2_ concentration of 400 μmol·mol^−1^, and leaf chamber relative humidity maintained at 60-70%. The experimental design consisted of three biological replicates arranged in a completely randomized configuration.

### 2.9 Statistical analysis

The statistical analyses were conducted using GraphPad Prism 8.0 (GraphPad Software, California, USA) and IBM SPSS Statistics 19 (IBM Corporation, New York, USA). Before analysis, data normality was verified using Shapiro-Wilk tests, while homogeneity of variance was confirmed through Levene’s test (*P>0.05*). All datasets satisfied the assumptions of normality and homoscedasticity. The results are shown as mean ± standard deviation (SD). A two-way analysis of variance (two-way ANOVA) followed by Tukey’s multiple comparisons test was employed to assess differences between groups, with a significance threshold set at *P < 0.05*.

## 3 Results

### 3.1 Multiple-sequence alignments and phylogenetic analysis

**Figure 1:**
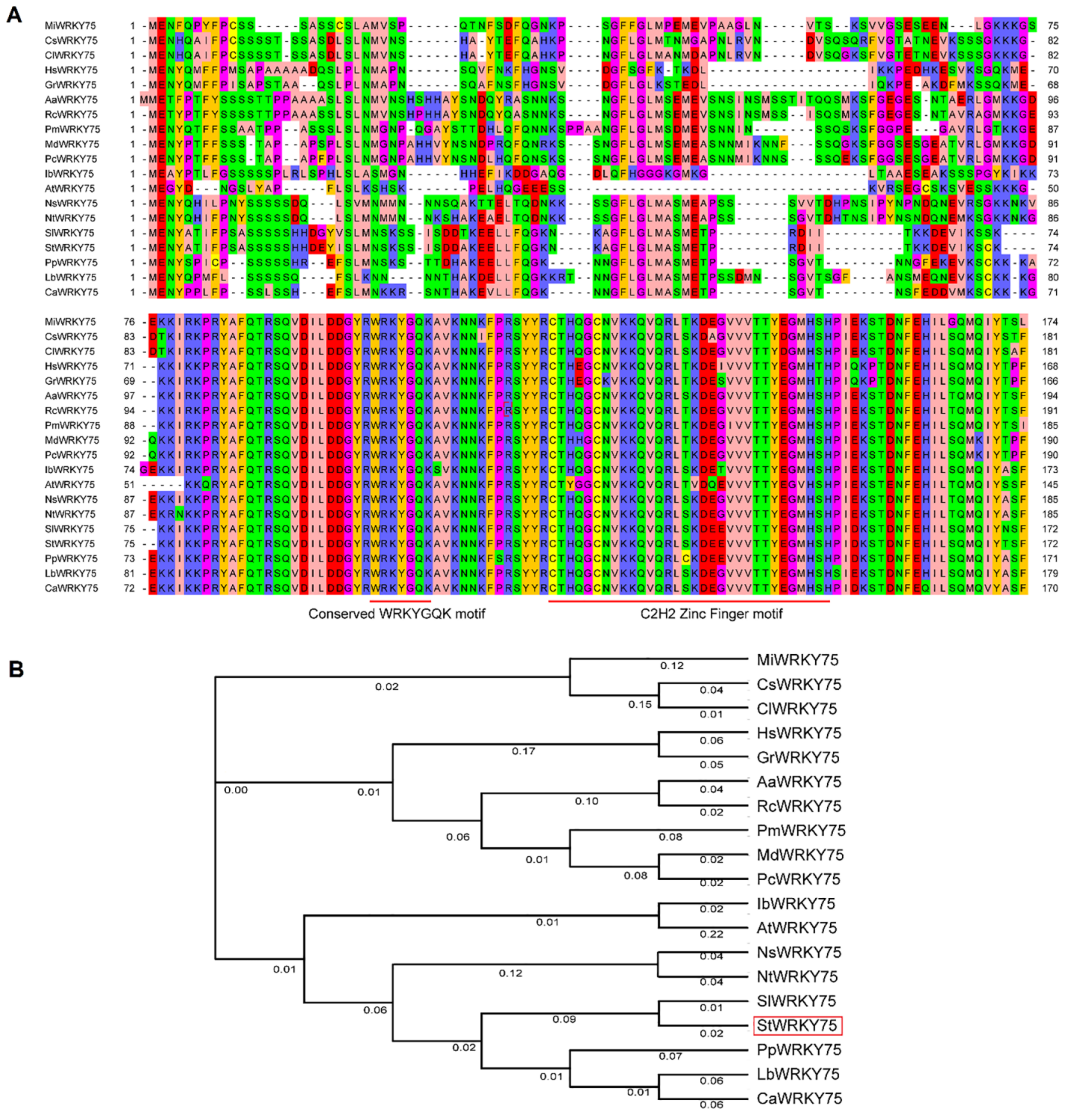
Multiple sequence alignment and phylogenetic relationships of StWRKY75 with other plant species. **(A)** Multiple sequence alignment of WRKY75 homologous proteins in potato and other plants. The different background colors represent a similar degree of amino acid sequences. The first red line indicates the WRKYGQK motif, and the second indicates the conserved C2H2 zinc-finger motif. **(B)** Neighbor-joining phylogenetic tree of the WRKY75 in potato and other plant species. The unrooted tree was generated using the MEGA 4.1 program with the neighbor-joining method. Bootstrap values (above 50%) from 1000 replicates are indicated at each branch.

The amino acid sequence alignment (**Figure 1A**) and phylogenetic tree (**Figure 1B**) demonstrate the evolutionary conservation of StWRKY75 among 18 other plant species. Mango (*Mangifera indica*) MiWRKY75, tea plant (*Camellia sinensis*) CsWRKY75, oil-seed camellia (*Camellia lanceoleosa*) ClWRKY75, Shrub althea (*Hibiscus syriacus*) HsWRKY75, peruvian cotton (*Gossypium raimondii*) GrWRKY75, silverweed (*Argentina anserina*) AaWRKY75, Chinese rose (*Rosa chinensis*) RcWRKY75, Japanese plum (*Prunus mume*) PmWRKY75, apple (*Malus domestica)* MdWRKY75, common pear (*Pyrus communis*) PcWRKY75, sweet potato (*Ipomoea batatas*) IbWRKY75, *Arabidopsis* (*Arabidopsis thaliana*) AtWRKY75, flowering tobacco (*Nicotiana sylvestris*) NsWRKY75, wild tobacco (*Nicotiana tomentosiformis*) NtWRKY75, tomato (*Solanum lycopersicum*) SlWRKY75, potato (*Solanum tuberosum*) StWRKY75, ground cherry (*Physalis pubescens*) PpWRKY75, wolfberry (*Lycium barbarum*) LbWRKY75, pepper (*Capsicum annuum*) CaWRKY75 and their predicted amino acid residue alignment is shown in **Figure 1A**. The NCBI Protein BLAST tool (https://blast.ncbi.nlm.nih.gov/Blast.cgi?PAGE=Proteins) was employed to retrieve the protein accession numbers of WRKY75 orthologs from diverse plant species, as listed in **Supplementary Table S1**. The sequence alignment analysis revealed that StWRKY75 shares significant sequence homology with orthologous proteins from diverse species, including *Solanum lycopersicum* (SlWRKY75), *Prunus mume* (PmWRKY75), *Rosa chinensis* (RcWRKY75), *Actinidia arguta* (AaWRKY75), *Gossypium raimondii* (GrWRKY75), and *Hibiscus syriacus* (HsWRKY75). All these proteins contain the conserved signature WRKYGQK at the N-terminus, followed by the C2H2 zinc-finger motif (CX_5_-C-X_23_-H-X_1_-H), which is characteristic of group II (**Figure 1A**). This conserved domain architecture confirms the evolutionary preservation of core functional foundations across these orthologs. Phylogenetic analysis revealed distinct clustering patterns among WRKY75 orthologs (**Figure 1B**). Notably, StWRKY75 exhibits the highest sequence homology with SlWRKY75, as they are clustered together in the phylogenetic tree. Members within each cluster exhibit conserved functional domains and significant evolutionary homology, suggesting conserved functional roles among phylogenetically related orthologs.

### 3.2 Expression profile of *StWRKY75* in potato under various abiotic stress treatments

**Figure 2:**
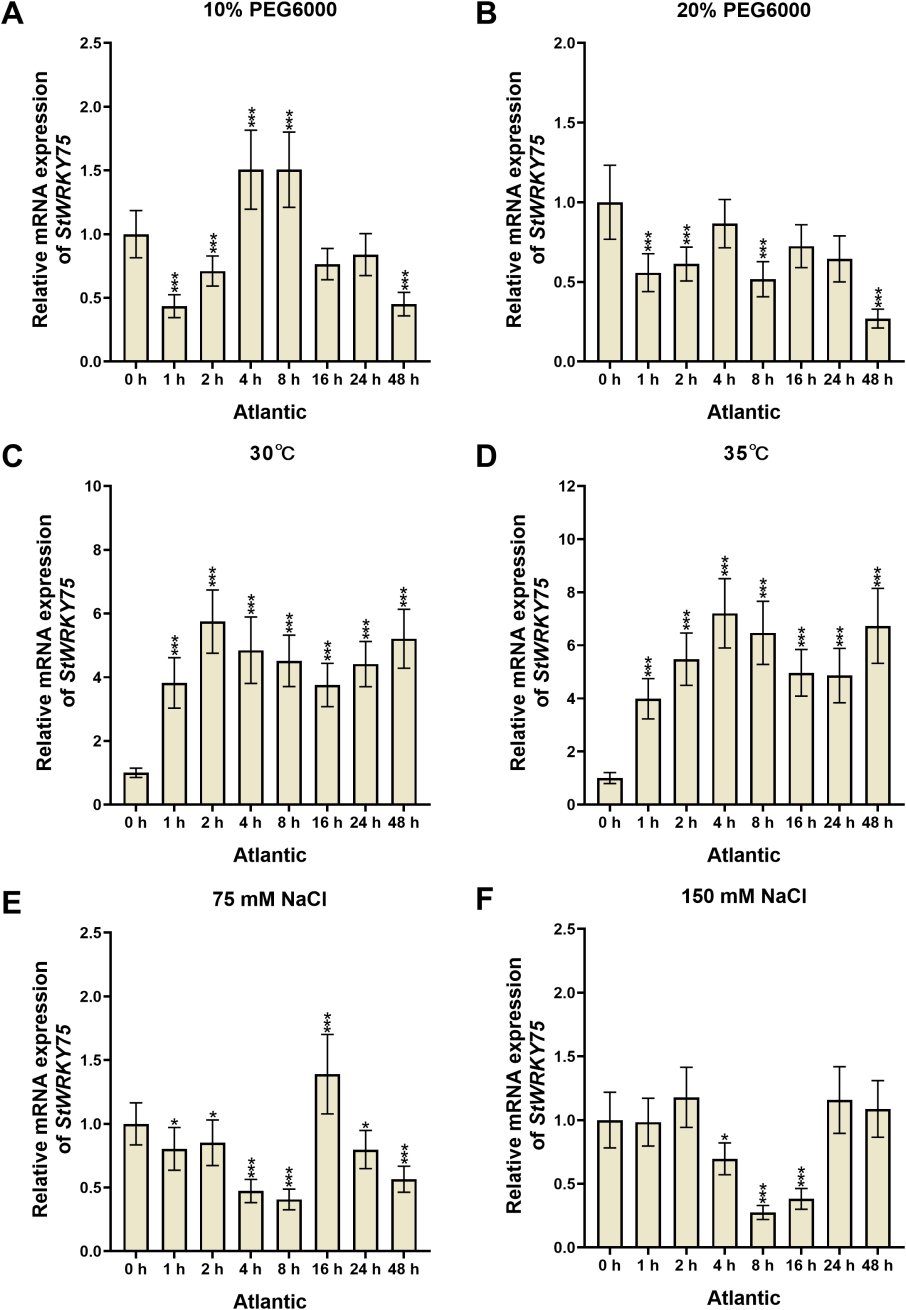
The expression patterns of *StWRKY75* in the leaves of potato cultivar ‘*Atlantic*’ in response to drought (10% and 20% PEG6000), heat (30°C and 35°C), and salinity (75 mM and 150 mM NaCl), induced at the time intervals of 0, 1, 2, 4, 8, 16, 24, and 48 h. The data are presented as mean ± standard deviation. P-values (*P < 0.05, ***P < 0.001) were calculated through ordinary two-way ANOVA followed by Tukey’s multiple comparisons test with a sample size of n = 9.

**Figure 3:**
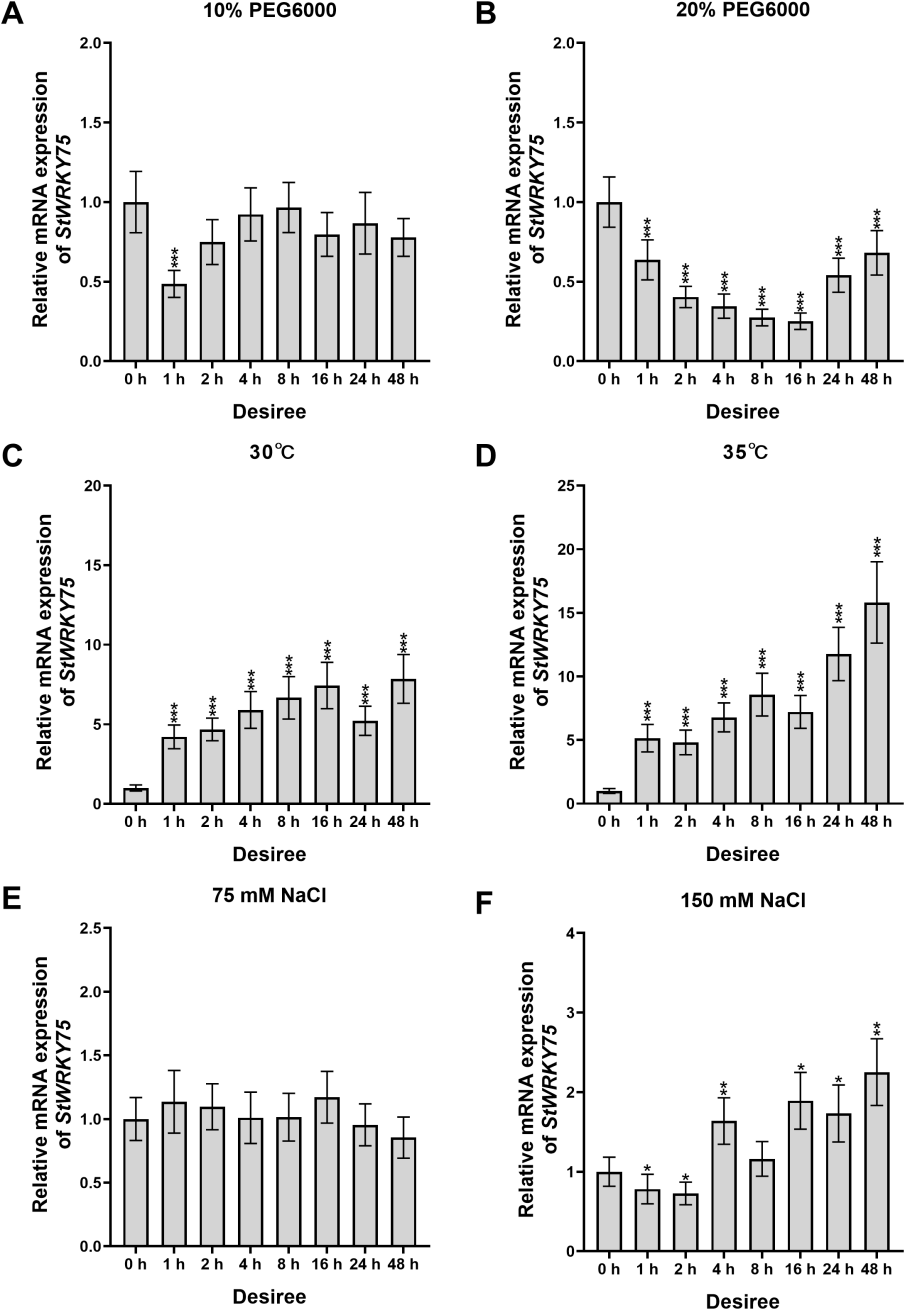
The expression patterns of *StWRKY75* in the leaves of potato cultivar ‘*Desiree*’ in response to drought (10% and 20% PEG6000), heat (30°C and 35°C), and salinity (75 mM and 150 mM NaCl), induced at the time intervals of 0, 1, 2, 4, 8, 16, 24, and 48 h. The data are presented as mean ± standard deviation. P-values (*P < 0.05, **P < 0.01, ***P < 0.001) were calculated through ordinary two-way ANOVA followed by Tukey’s multiple comparisons test with a sample size of n = 9.

To elucidate the transcriptional dynamics of *StWRKY75* under various abiotic stresses, this study systematically analyzed the relative expression patterns in leaves of potato cultivars ‘*Desiree*’ and ‘*Atlantic*’ using qRT-PCR (**Figures 2**, **3**). In ‘*Desiree*’, *StWRKY75* expression showed continuous upregulation from 1 to 48 hours post-treatment (hpt) under 30°C (peaking at 48 hpt), while 35°C stress treatment induced a sustained upregulated expression pattern reaching maximum at 48 hpt (**Figures 3C, D**). ‘*Atlantic*’ displayed significant upregulation from 1–48 hpt under 30°C (peak at 2 hpt), with 35°C stress treatment triggering rapid response (peak at 4 hpt), indicating the gene’s involvement in thermotolerance regulation through rapid response to heat stress (**Figures 2C, D**). Under drought stress, ‘*Desiree*’ showed insensitivity to 10% PEG6000 (only significant downregulation at 1 hpt), whereas 20% PEG6000 treatment caused continuous suppression from 1–48 hpt (minimum at 16 hpt), suggesting its potential negative regulatory role in severe drought response (**Figures 3A, B**). ‘*Atlantic*’ exhibited significant upregulation at 4–8 hpt (peaks at 4 and 8 hpt) but downregulation at other time points (minimum at 1 hpt) under 10% PEG6000, with 20% PEG6000 inducing overall suppression (minimum at 48 hpt) (**Figures 2A, B**). Salt stress responses showed cultivar-specific patterns: ‘*Desiree*’ showed no significant changes under 75 mM NaCl but significant upregulation at 4 hpt (peak at 48 hpt) under 150 mM NaCl (**Figures 3E, F**); ‘*Atlantic*’ displayed only transient upregulation at 16 hpt under 75 mM NaCl, while 150 mM NaCl caused significant suppression from 4–16 hpt (minimum at 8 hpt) (**Figures 2E, F**). These findings demonstrate that *StWRKY75* functions as a core regulator in heat stress response with rapid activation characteristics under both 30°C and 35°C stress treatments, while its roles in drought and salt stress may be differentially modulated by cultivar-specific genetic backgrounds and stress intensity.

### 3.3 Subcellular localization analysis and construction of *StWRKY75* transgenic potato plants

**Figure 4:**
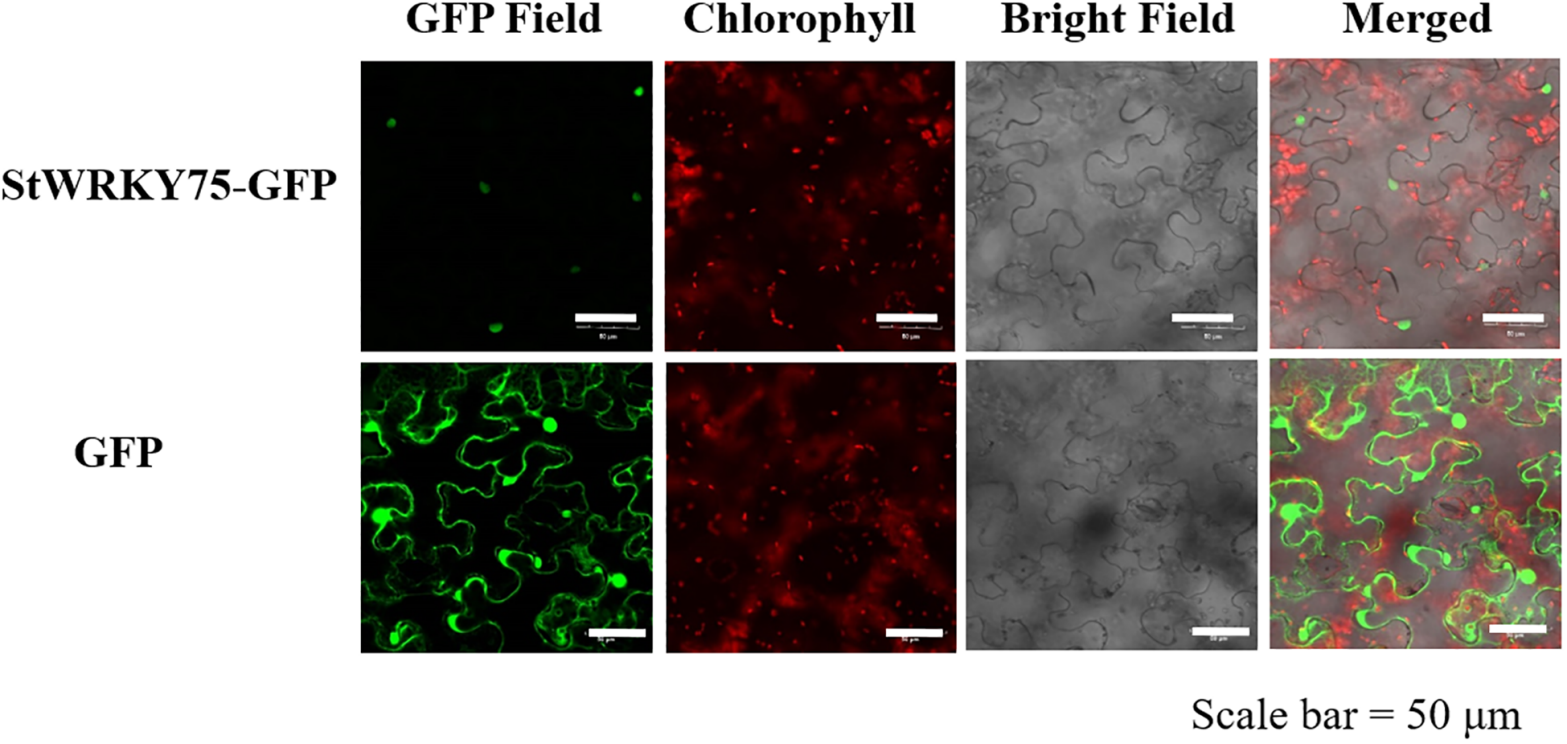
Subcellular localization of StWRKY75-GFP fusion protein. Confocal scanning laser microscopy analysis of tobacco transformed with pCAM35-GFP-StWRKY75. The empty vector (GFP) served as a control. Bar = 50 μm.

To assess the subcellular localization of StWRKY75 TF, tobacco leaves were infiltrated with *Agrobacterium tumefaciens* strain GV3101 carrying the recombinant plasmid pCAM35S-GFP-StWRKY75 and the empty vector control (pCAM35S-GFP). Laser confocal microscopy showed that the StWRKY75-GFP fusion protein localized exclusively to the nucleus by green protein signals (**Figure 4**). In contrast, the GFP vector (control) was distributed in the plasma membrane, cytoplasm, and nucleus. These results demonstrate the nuclear-specific localization of StWRKY75.

To functionally characterize *StWRKY75* in potato, we established transgenic overexpression (OE) and RNA interference (RNAi) lines in two commercial cultivars, ‘*Atlantic*’ and ‘*Desiree*’. The qRT-PCR analysis revealed that *StWRKY75* transcript levels were significantly elevated in all OE lines (OE-1 to OE-8; *** P < 0.001, **Supplementary Figure S1**), whereas its expression was markedly suppressed in RNAi lines (RNAi-1 to RNAi-8; * * *P < 0.001, **Supplementary Figure S1**). Based on expression profiling, we selected three most representative lines from each group for subsequent functional studies: OE-3, OE-6, and OE-7 (showing highest overexpression) along with RNAi-1, RNAi-4, and RNAi-5 (exhibiting strongest suppression) in ‘*Atlantic*’(* * *P < 0.001, **Supplementary Figures S1A, B**); similarly, OE-1, OE-2, OE-4 and RNAi-1, RNAi-3, RNAi-4 were chosen from ‘*Desiree*’ lines (* * *P < 0.001, **Supplementary Figures S1C, D**). These selected transgenic lines were subsequently employed to investigate the functional role of *StWRKY75* in thermotolerance responses.

### 3.4 *StWRKY75* regulates the growth and biomass of potato plants under heat stress

Under optimal growth conditions (20°C), transgenic and NT control lines of potato cultivars ‘*Atlantic*’ and ‘*Desiree*’ exhibited comparable performance (*P>0.05*) in all evaluated growth parameters, including plant height, tuber weight per plant, fresh weight, dry weight, root fresh weight, and root dry weight (**Table 1**). When exposed to 30°C heat stress, all genotypes of both cultivars exhibited significant reductions in growth metrics (*P<0.05, **P<0.01). However, *StWRKY75*-overexpressing lines in both ‘*Atlantic*’ and ‘ *Desiree*’ demonstrated enhanced thermotolerance, with significantly greater plant height, tuber yield, and biomass accumulation compared to NT and RNAi lines (**Table 1**). Conversely, RNAi lines showed pronounced growth inhibition, with all measured parameters significantly lower than NT controls (*P<0.05, **P<0.01). At 35°C, heat stress, growth parameters declined more severely across all genotypes, with heat stress severely impeding potato growth. Nevertheless, OE lines in both cultivars maintained significantly superior growth performance (**Table 1**), while RNAi lines suffered exacerbated heat stress damage, displaying further significant reductions in biomass and tuber yield compared to NT plants (*P<0.05, **P<0.01, ***P<0.001). These findings collectively indicate that *StWRKY75* overexpression confers thermotolerance by mitigating heat-induced growth suppression, while its knockdown exacerbates sensitivity, underscoring the gene’s critical role in potato heat stress adaptation.

**Table 1:** Effects of *StWRKY75* on potato growth indicators in response to heat treatment.

Heat stress (potato cultivar)	Genotype	Plant height (cm)	Tuber yield per plant (g)	Fresh weight (g)	Dry weight (g)	Root fresh weight (g)	Root dry weight (g)
20°C (*Atlantic*)	NT	52.73 ± 11.08	20.52 ± 3.47	174.62 ± 33.61	24.74 ± 3.77	14.98 ± 2.61	1.66 ± 0.32
	OE-3	52.71 ± 11.29 ns	21.19 ± 3.2 ns	176.43 ± 29.26 ns	25.25 ± 4.35 ns	15.26 ± 3.37 ns	1.68 ± 0.32 ns
	OE-6	53.39 ± 8.96 ns	21.55 ± 3.37 ns	175.23 ± 31.85 ns	24.76 ± 3.99 ns	15.5 ± 2.71 ns	1.63 ± 0.31 ns
	OE-7	53.57 ± 14.03 ns	21.5 ± 3.46 ns	175.49 ± 34.16 ns	24.82 ± 3.33 ns	15.13 ± 2.93 ns	1.63 ± 0.28 ns
	RNAi-1	52.6 ± 10.29 ns	20.63 ± 3.14 ns	171.11 ± 31.87 ns	23.3 ± 4.6 ns	15.05 ± 3.4 ns	1.6 ± 0.27 ns
	RNAi-4	50.95 ± 9.92 ns	20.85 ± 3.77 ns	174.95 ± 27.33 ns	24.46 ± 4.46 ns	15.26 ± 2.65 ns	1.66 ± 0.26 ns
	RNAi-5	50.2 ± 8.88 ns	20.67 ± 4.04 ns	172.29 ± 29.35 ns	23.5 ± 5.41 ns	15.19 ± 3.51 ns	1.68 ± 0.26 ns
30°C (*Atlantic*)	NT	33.73 ± 6.28	8.32 ± 1.51	139.47 ± 27.22	17.69 ± 3.32	11.54 ± 2.22	1.06 ± 0.28
	OE-3	43.49 ± 6.54*	11.48 ± 2.17*	172.09 ± 23.31*	23.05 ± 4.03**	14.42 ± 2.4*	1.38 ± 0.25*
	OE-6	45.41 ± 6.52**	11.23 ± 1.7*	173.99 ± 42.3*	22.33 ± 4.5*	14.93 ± 3.07*	1.44 ± 0.27**
	OE-7	44.77 ± 7.93*	11.92 ± 1.9**	174.13 ± 32.9*	23.07 ± 4.59**	15.23 ± 2.55**	1.35 ± 0.33*
	RNAi-1	24.22 ± 5.18*	5.23 ± 1.06*	99.66 ± 17.54**	13.21 ± 3.08*	7.91 ± 1.74**	0.7 ± 0.12**
	RNAi-4	22.61 ± 4.35**	4.95 ± 0.77**	102.44 ± 12.82*	13.37 ± 2.25*	8.25 ± 1.71*	0.68 ± 0.19**
	RNAi-5	22.83 ± 5.8*	4.95 ± 0.86**	101.33 ± 15.62**	11.59 ± 2.71**	8.51 ± 1.42*	0.73 ± 0.12*
35°C (*Atlantic*)	NT	17.57 ± 3.23	1.71 ± 0.43	74.72 ± 16.18	11.89 ± 2.01	6.01 ± 1.14	0.64 ± 0.11
	OE-3	26.95 ± 4.01*	7.84 ± 0.65***	119.08 ± 19.55**	16.92 ± 2.79 *	9.65 ± 2.37**	1.06 ± 0.22***
	OE-6	28.70 ± 5.21**	6.61 ± 0.54*	125.44 ± 23.74***	17.29 ± 3.73**	10.57 ± 1.8***	0.99 ± 0.18**
	OE-7	26.82 ± 4.42*	6.82 ± 0.72*	118.82 ± 25.87**	16.81 ± 3.12 *	9.28 ± 2.12*	0.97 ± 0.15*
	RNAi-1	8.09 ± 2.07*	0.3 ± 0.07**	25.96 ± 4.64***	5.73 ± 0.73**	2.90 ± 0.45*	0.26 ± 0.06**
	RNAi-4	6.00 ± 1.96**	0.22 ± 0.04**	32.29 ± 5.68**	6.14 ± 0.64**	2.39 ± 0.46**	0.35 ± 0.06*
	RNAi-5	7.63 ± 1.87*	0.25 ± 0.04**	27.57 ± 6.53***	5.19 ± 0.86***	2.17 ± 0.46**	0.22 ± 0.09***
20°C (*Desiree*)	NT	47.77 ± 9.59	27.09 ± 3.19	172.63 ± 36.72	24.31 ± 3.53	22.54 ± 3.52	2.62 ± 0.49
	OE-3	48.83 ± 8.44 ns	28.26 ± 4.64 ns	173.14 ± 33.05 ns	25.05 ± 5.16 ns	23.11 ± 4.14 ns	2.95 ± 0.59 ns
	OE-6	48.62 ± 6.70 ns	27.69 ± 5.97 ns	171.41 ± 36.56 ns	25.05 ± 4.28 ns	23.88 ± 5.33 ns	2.86 ± 0.54 ns
	OE-7	47.61 ± 6.05 ns	28.86 ± 4.59 ns	169.03 ± 28.99 ns	24.38 ± 5.60 ns	23.37 ± 4.6 ns	2.89 ± 0.54 ns
	RNAi-1	47.24 ± 9.16 ns	27.13 ± 4.17ns	172.41 ± 30.61 ns	24.18 ± 3.52 ns	21.51 ± 4.62 ns	2.37 ± 0.49 ns
	RNAi-4	46.74 ± 9.15 ns	26.82 ± 2.60 ns	170.64 ± 29.17 ns	23.33 ± 4.33 ns	21.89 ± 3.50 ns	2.29 ± 0.45 ns
	RNAi-5	48.17 ± 9.39 ns	27.35 ± 4.81 ns	166.87 ± 25.91 ns	22.57 ± 4.04 ns	21.6 ± 3.61 ns	2.24 ± 0.38 ns
30°C (*Desiree*)	NT	33.75 ± 6.34	9.02 ± 1.77	124.84 ± 20.07	17.98 ± 3.29	14.98 ± 2.61	1.71 ± 0.32
	OE-3	42.53 ± 7.24*	13.35 ± 2.04**	157.41 ± 31.68*	22.82 ± 4.54*	20.62 ± 3.47**	2.26 ± 0.48*
	OE-6	43.89 ± 8.37**	12.75 ± 2.57*	159.25 ± 26.68*	22.39 ± 3.89*	19.04 ± 3.39*	2.28 ± 0.44**
	OE-7	42.64 ± 7.97*	13.09 ± 2.22*	168.50 ± 27.56**	23.86 ± 4.90**	19.75 ± 3.53**	2.24 ± 0.45*
	RNAi-1	24.50 ± 5.52*	4.91 ± 0.87*	87.41 ± 19.34**	11.74 ± 2.37*	10.49 ± 1.88*	1.20 ± 0.24*
	RNAi-4	22.20 ± 4.69**	5.37 ± 1.02*	89.25 ± 16.55*	12.40 ± 2.49**	10.32 ± 2.19**	1.23 ± 0.20*
	RNAi-5	23.98 ± 5.31*	4.75 ± 0.89**	93.36 ± 15.23*	12.88 ± 3.06*	10.94 ± 1.71*	1.07 ± 0.21**
35°C (*Desiree*)	NT	22.75 ± 5.25	4.36 ± 0.69	81.87 ± 16.79	11.72 ± 2.15	9.42 ± 1.75	1.04 ± 0.18
	OE-3	34.30 ± 5.15**	9.93 ± 1.34***	127.04 ± 18.97***	16.10 ± 2.83*	13.33 ± 2.70*	1.51 ± 0.28*
	OE-6	32.16 ± 6.11*	8.94 ± 1.34*	115.22 ± 17.87*	17.05 ± 3.35**	14.17 ± 2.57**	1.69 ± 0.38**
	OE-7	31.01 ± 5.22*	8.58 ± 1.19*	119.86 ± 27.47**	18.86 ± 3.12***	15.26 ± 2.21***	1.75 ± 0.31***
	RNAi-1	9.09 ± 3.19***	0.45 ± 0.08**	50.16 ± 8.81*	5.98 ± 1.38**	5.47 ± 1.13*	0.55 ± 0.11*
	RNAi-4	13.57 ± 3.23*	0.59 ± 0.12**	49.31 ± 8.57*	6.15 ± 1.31**	4.22 ± 1.05**	0.45 ± 0.12**
	RNAi-5	12.63 ± 2.65**	0.74 ± 0.10*	42.18 ± 9.45**	7.05 ± 1.21*	4.74 ± 1.12**	0.33 ± 0.11***

*StWRKY75* modulates growth parameters in potato cultivars ‘*Atlantic*’ and ‘*Desiree*’ under different temperature conditions (20°C, 30°C, and 35°C). In the ‘*Atlantic*’ cultivar: NT, non-transgenic plants; OE, pBI121-EGFP-StWRKY75-transgenic plants (OE-3, OE-6, and OE-7); RNAi, pART-StWRKY75-RNAi-transgenic plants (RNAi-1, RNAi-4 and RNAi-5). In the ‘*Desiree*’ cultivar: NT, non-transgenic plants; OE, pBI121-EGFP-StWRKY75-transgenic plants (OE-1, OE-2, and OE-4); RNAi, pART-StWRKY75-RNAi-transgenic plants (RNAi-1, RNAi-3 and RNAi-4). The data are presented as mean ± standard deviation. P-values (*P < 0.05, **P < 0.01, ***P < 0.001) were calculated through ordinary two-way ANOVA followed by Tukey’s multiple comparisons test with a sample size of n = 9.

### 3.5 *StWRKY75* modulates antioxidant enzymes (APX, POD, CAT, SOD) activities, MDA and proline contents in potatoes under heat stress

**Figure 5:**
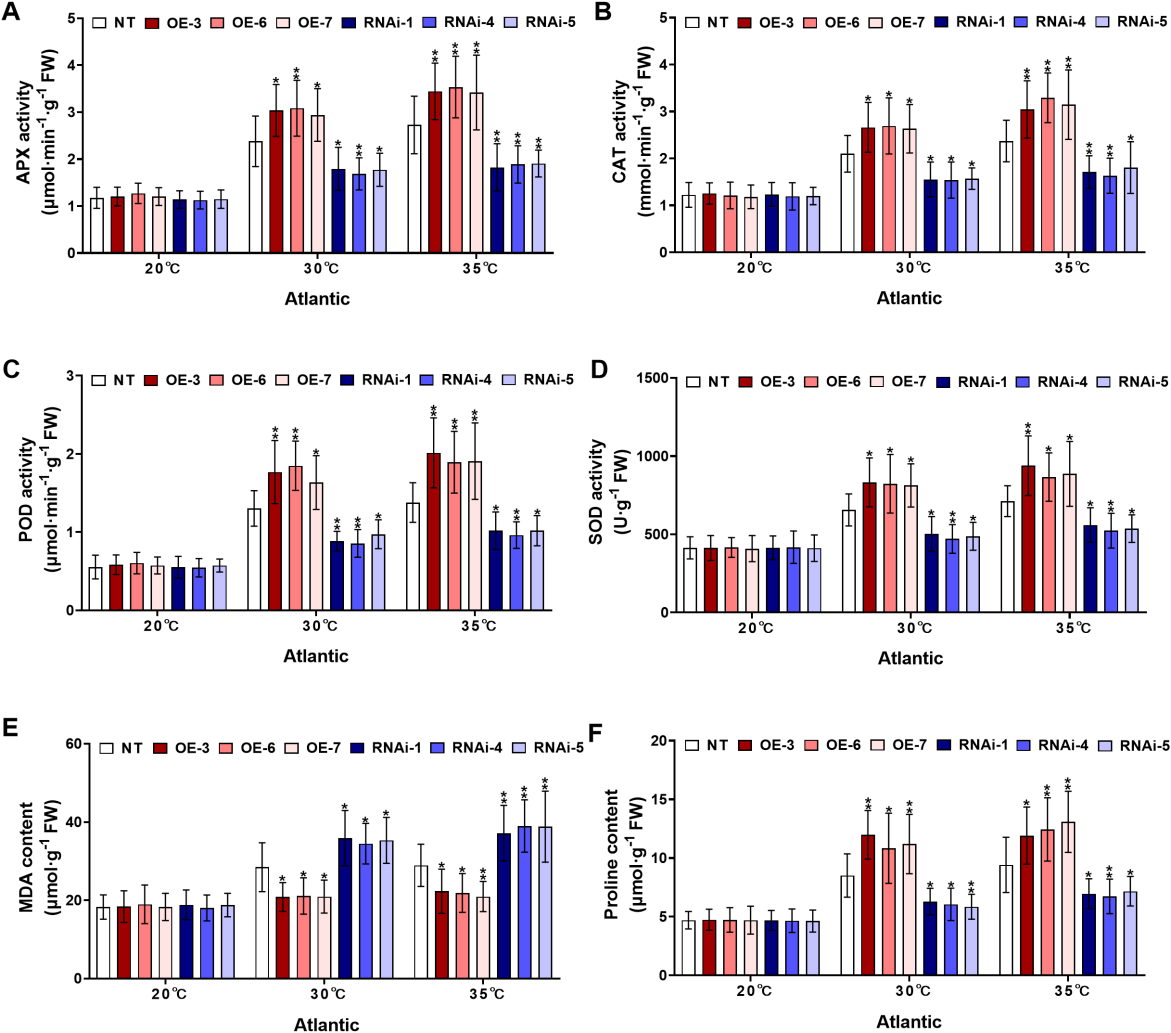
*StWRKY75* modulates potato plants of cultivar ‘*Atlantic*’; **(A)** APX, **(B)** CAT, **(C)** POD, **(D)** SOD, **(E)** MDA, and **(F)** proline, after exposure to 20°C, 30°C, and 35°C of heat stress. NT, non-transgenic plants; OE, pBI121-EGFP-StWRKY75-transgenic plants (OE-3, OE-6, and OE-7); RNAi, pART-StWRKY75-RNAi-transgenic plants (RNAi-1, RNAi-4 and RNAi-5). The data are presented as mean ± standard deviation. P-values (*P < 0.05, **P < 0.01) were calculated through ordinary two-way ANOVA followed by Tukey’s multiple comparisons test with a sample size of n = 9.

To assess the hypothesis that *StWRKY75*-overexpressed potato plants of cultivars ‘*Atlantic*’ and *‘Desiree’* regulate antioxidant metabolism under heat stress, the activities of key antioxidant enzymes, including APX, CAT, POD, and SOD along with the oxidative stress marker MDA and the non-enzymatic osmolyte proline were investigated under varying temperature stress treatments (20°C, 30°C, and 35°C). Under control conditions (20°C), no significant differences were observed in APX, CAT, POD, or SOD activities, MDA levels, or proline content between transgenic (*StWRKY75*-overexpressing and RNAi) and NT plants of the potato cultivar ‘*Atlantic*’ (**Figures 5A-F**). However, when subjected to 30°C heat stress, the *StWRKY75*-overexpressing lines (OE-3, OE-6, OE-7) exhibited significantly enhanced activities of APX (**Figure 5A**), CAT (**Figure 5B**), POD (**Figure 5C**), and SOD (**Figure 5D**), along with increased proline accumulation (**Figure 5F**). In contrast, RNAi knockdown lines (RNAi-1, RNAi-4, RNAi-5) showed marked reductions in these parameters compared to NT controls. Notably, MDA content, a key indicator of oxidative damage, was significantly lower in *StWRKY75*-overexpressing plants (**Figure 5E**), whereas RNAi lines accumulated higher MDA levels, indicating greater susceptibility to oxidative stress. Under more severe heat stress (35°C), *StWRKY75*-overexpressing plants showed significantly greater increases in the activities of APX (**Figure 5A**), CAT (**Figure 5B**), POD (**Figure 5C**), and SOD (**Figure 5D**) activities, along with significantly elevated proline content (**Figure 5E**), when compared with NT control plants. Conversely, RNAi lines exhibited further suppression of these physiological indices. The *StWRKY75*-overexpressing lines maintained lower MDA accumulation (**Figure 5F**) at 35°C, demonstrating reduced oxidative damage, while RNAi lines showed exacerbated membrane lipid peroxidation due to impaired stress tolerance.

**Figure 6:**
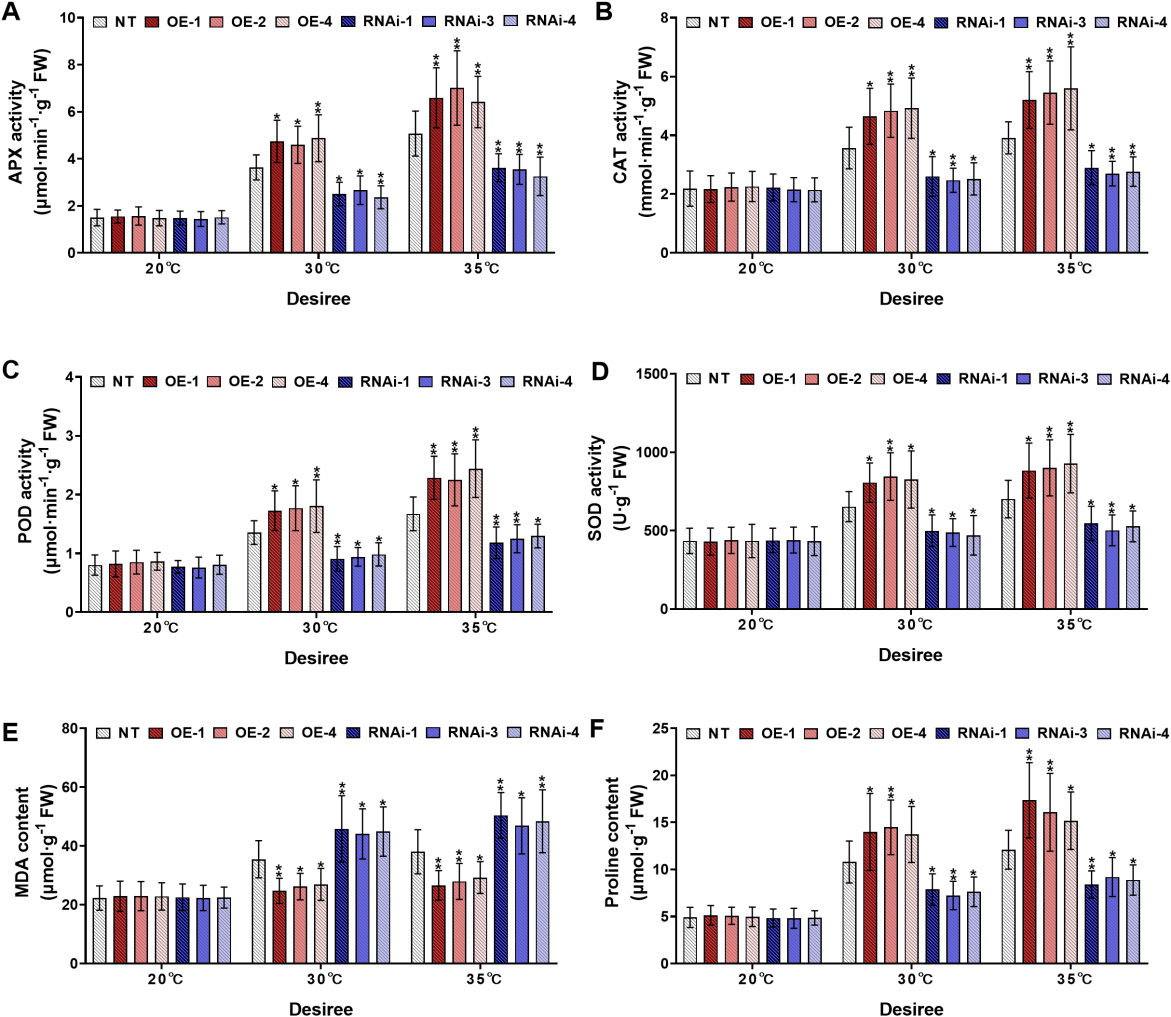
*StWRKY75* modulates potato plants of cultivar ‘*Desiree*’; **(A)** APX, **(B)** CAT, **(C)** POD, **(D)** SOD, **(E)** MDA, and **(F)** proline, after exposure to 20°C, 30°C, and 35°C of heat stress. NT, non-transgenic plants; OE, pBI121-EGFP-StWRKY75-transgenic plants (OE-1, OE-2, and OE-4); RNAi, pART-StWRKY75-RNAi-transgenic plants (RNAi-1, RNAi-3 and RNAi-4). The data are presented as mean ± standard deviation. P-values (*P < 0.05, **P < 0.01) were calculated through ordinary two-way ANOVA followed by Tukey’s multiple comparisons test with a sample size of n = 9.

Similarly, the cultivar *‘Desiree’* displayed comparable responses under high-temperature stress treatments (30°C and 35°C). Under control conditions (20°C), no significant differences were observed among all experimental potato plants for different physiological parameters (**Figures 6A-F**). However, when heat stress treatment was increased (30°C), the *StWRKY75*-overexpressing lines (OE-1, OE-2, OE-4) exhibited significantly increased activities of APX (**Figure 6A**), CAT (**Figure 6B**), POD (**Figure 6C**), and SOD (**Figure 6D**), and higher proline accumulation (**Figure 6F**). In contrast, RNAi-silenced lines (RNAi-1, RNAi-3, RNAi-4) showed a pronounced reduction in these physiological markers. Additionally, the MDA content was significantly lower in *StWRKY75*-overexpressing lines (**Figure 6E**), whereas RNAi lines exhibited higher MDA levels compared to NT control lines. Under severe heat stress conditions (35°C), all experimental lines exhibited exacerbated physiological damage. However, the *StWRKY75*-overexpressing line demonstrated significantly enhanced enzymatic activity of key antioxidant enzymes, including APX (**Figure 6A**), CAT (**Figure 6B**), POD (**Figure 6C**), and SOD (**Figure 6D**), as well as elevated proline level (**Figure 6F**). In contrast, *StWRKY75* RNA interference (RNAi) lines exhibited a noticeable decline in these physiological parameters compared to NT plants. Likewise, the *StWRKY75*-overexpressing lines showed declined MDA levels (**Figure 6F**), and the opposite was true for *StWRKY75*-knockdown lines than those in NT potato lines. These findings suggest that *StWRKY75* plays a critical regulatory role in enhancing thermotolerance by modulating antioxidant defense mechanisms and osmoprotectant synthesis under extreme heat stress conditions.

### 3.6 *StWRKY75* regulates the stress-responsive genes in potatoes under heat stress

To investigate the genetic regulatory networks and biochemical mechanisms underlying heat stress adaptation in two distinct potato cultivars, ‘*Atlantic*’ and ‘*Desiree*’, this study systematically analyzed the expression profiles of key stress-responsive genes (including *StAPX*, *StCAT*, *StSOD*, *StPOD*, *StP5CS*, *StHSP20*, *StHSP70*, and *StHSP90*) across all transgenic and NT lines under different heat stress treatments (20°C, 30°C, and 35°C). The research aimed to comprehensively evaluate the differential regulation of antioxidant enzyme-related genes, proline biosynthesis-related genes, and heat shock-related genes under escalating temperature stress, thereby elucidating the genetic basis of *StWRKY75*-mediated thermotolerance.

**Figure 7:**
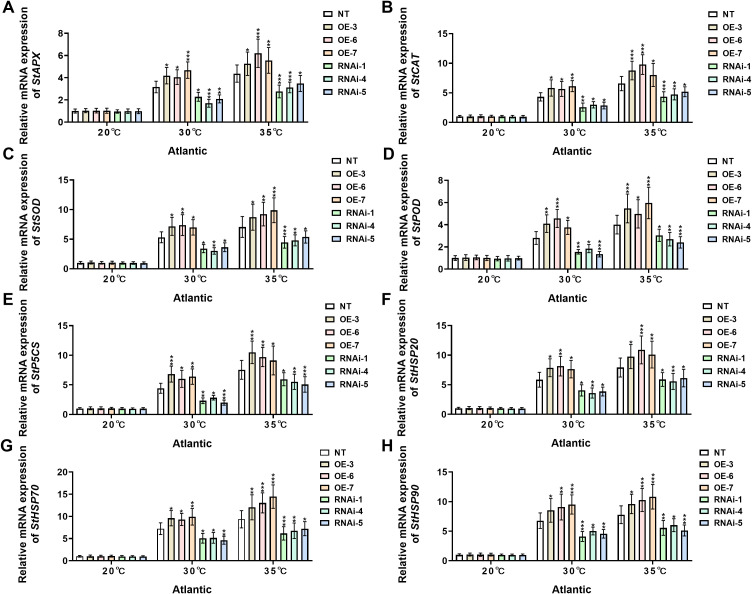
*StWRKY75* regulates the relative mRNA expression of potato plants of cultivar ‘*Atlantic*’; **(A)** *StAPX*, **(B)** *StCAT*, **(C)** *StSOD*, **(D)** *StPOD*, **(E)** *StP5CS*, **(F)** *StHSP20*, **(G)** *StHSP70*, and **(H)** *StHSP90*, after exposure to 20°C, 30°C, and 35°C of heat stress. NT, non-transgenic plants; OE, pBI121-StWRKY75-transgenic plants (OE-3, OE-6, and OE-7); RNAi, pART-StWRKY75-RNAi-transgenic plants (RNAi-1, RNAi-4, and RNAi-5). The data are presented as mean ± standard deviation. P-values (*P < 0.05, **P < 0.01, ***P < 0.001) were calculated through ordinary two-way ANOVA followed by the Tukey’s multiple comparisons test with a sample size of n = 9.

**Figure 8:**
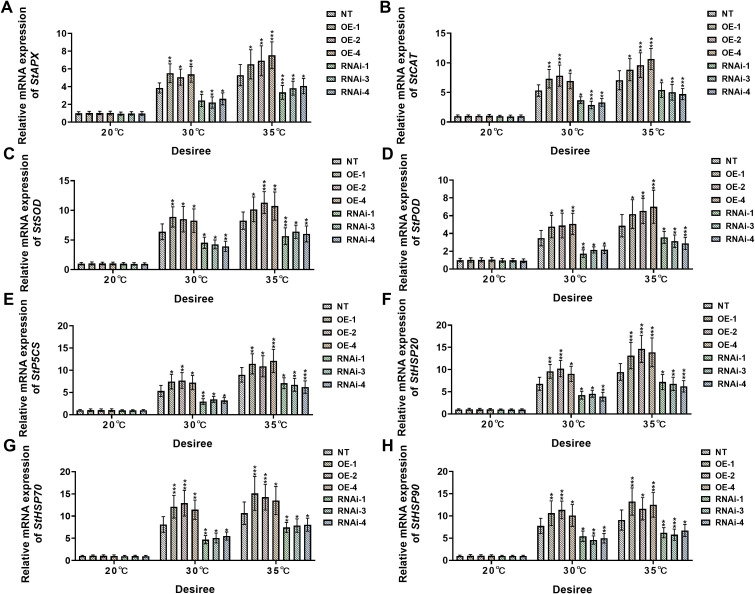
*StWRKY75* regulates the relative mRNA expression of potato plants of cultivar ‘*Desiree*’; **(A)** *StAPX*, **(B)** *StCAT*, **(C)** *StSOD*, **(D)** *StPOD*, **(E)** *StP5CS*, **(F)** *StHSP20*, **(G)** *StHSP70*, and **(H)** *StHSP90*, after exposure to 20°C, 30°C, and 35°C of heat stress. NT, non-transgenic plants; OE, pBI121-StWRKY75-transgenic plants (OE-1, OE-2, and OE-4); RNAi, pART-StWRKY75-RNAi-transgenic plants (RNAi-1, RNAi-3, and RNAi-4). The data are presented as mean ± standard deviation. P-values (*P < 0.05, **P < 0.01, ***P < 0.001) were calculated through ordinary two-way ANOVA followed by the Tukey’s multiple comparisons test with a sample size of n = 9.

Under control conditions (20°C), no significant differential expression was observed for any of the assessed stress-responsive genes (*StAPX*, *StCAT*, *StSOD*, *StPOD*, *StP5CS*, *StHSP20*, *StHSP70*, and *StHSP90*) in ‘*Atlantic*’ potato plants (**Figures 7A-H**). However, when temperature was increased to 30°C, *StWRKY75*-overexpressing plants (OE-3, OE-6, OE-7) showed significant upregulation in transcript levels of *StAPX* (**Figure 7A**), *StCAT* (**Figure 7B**), *StSOD* (**Figure 7C**), *StPOD* (**Figure 7D**), *StP5CS* (**Figure 7E**), *StHSP20* (**Figure 7F**), *StHSP70* (**Figure 7G**), and *StHSP90* (**Figure 7H**), while *StWRKY75* RNA interference lines (RNAi-1, RNAi-4, RNAi-5) exhibited marked downregulation compared to NT controls. This differential expression pattern became more pronounced under 35°C heat stress, with *StWRKY75*-overexpressing lines showing extremely significant upregulation of these stress-responsive genes relative to NT plants. Conversely, all target gene expressions were suppressed in *StWRKY75* knockdown plants. Similarly, under standard growth conditions (20°C), the ‘*Desiree*’ cultivar maintained stable transcriptional levels of these key stress-responsive genes across NT and transgenic lines without significant differences (**Figures 8A-H**). At 30°C heat stress, however, *StWRKY75*-overexpressing lines (OE-1, OE-2, OE-4) demonstrated significant upregulation of antioxidant genes [*StAPX* (**Figure 8A**), *StCAT* (**Figure 7B**), *StSOD* (**Figure 7C**), *StPOD* (**Figure 8D**)], the proline biosynthesis gene *StP5CS* (**Figure 8E**), and heat shock protein genes [*StHSP20* (**Figure 8F**), *StHSP70* (**Figure 8G**), *StHSP90* (**Figure 8H**)]. In contrast, these stress-responsive genes were significantly suppressed in *StWRKY75* knockdown lines (RNAi-1, RNAi-3, RNAi-4) compared to NT plants. Elevated temperatures intensified these transcriptional responses, with the most prominent differential expression observed at 35°C. At this threshold, *StWRKY75*-overexpressing plants maintained strong upregulation of all stress-related genes (**Figures 8A-H**), while RNAi lines showed progressively declining expression profiles. These findings robustly demonstrate that *StWRKY75* functions as a central transcriptional regulator during thermal adaptation, coordinating the upregulation of these genes (*StAPX*, *StCAT*, *StSOD*, *StPOD*, *StP5CS*, *StHSP20*, *StHSP70*, and *StHSP90*) to enhance potato thermotolerance.

### 3.7 *StWRKY75* regulates gas exchange traits of potatoes under heat stress

**Figure 9:**
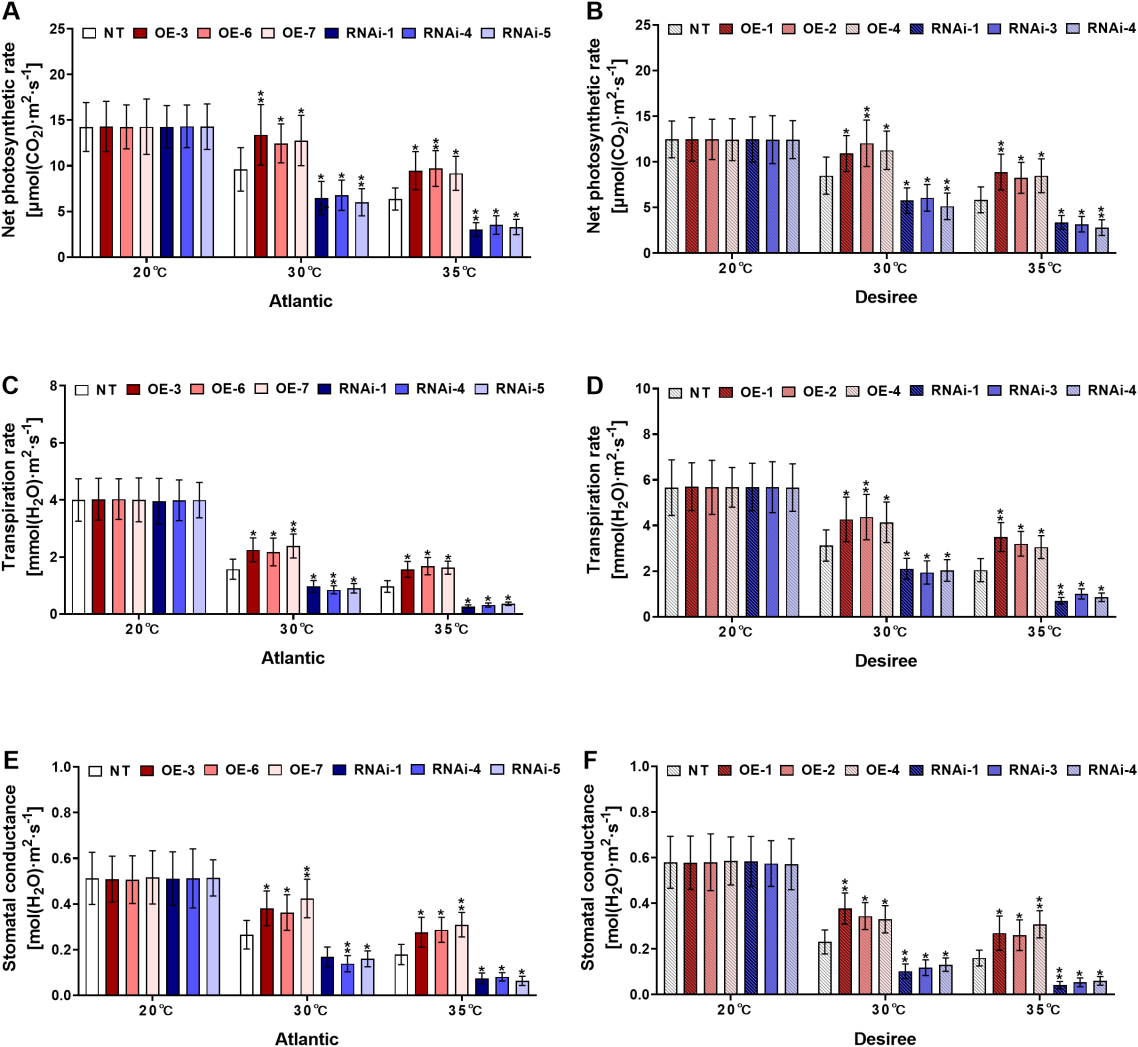
*StWRKY75* regulates the gas exchange parameters of potato plants of cultivars, ‘*Atlantic*’ and ‘*Desiree*’; **(A, B)** photosynthetic rates, **(C, D)** transpiration rates, and **(E, F)** stomatal conductance, respectively, after exposure to 20°C, 30°C, and 35°C of heat stress. In the ‘*Atlantic*’ cultivar: NT: non-transgenic plants; OE, pBI121-EGFP-StWRKY75-transgenic plants (OE-3, OE-6, and OE-7); RNAi, pART-StWRKY75-RNAi-transgenic plants (RNAi-1, RNAi-4 and RNAi-5). In the ‘*Desiree*’ cultivar: NT: non-transgenic plants; OE, pBI121-EGFP-StWRKY75-transgenic plants (OE-1, OE-2, and OE-4); RNAi, pART-StWRKY75-RNAi-transgenic plants (RNAi-1, RNAi-3 and RNAi-4).The data are presented as mean ± standard deviation. P-values (*P < 0.05, **P < 0.01) were calculated through ordinary two-way ANOVA followed by the Tukey’s multiple comparisons test with a sample size of n = 9.

Under high-temperature stress regimes (30°C and 35°C), key photosynthetic parameters, including Pn, Gs, and E, were measured. Under control conditions (20°C), no significant differences were observed in gas exchange parameters between transgenic and NT plants of both ‘*Atlantic*’ and ‘*Desiree*’ potato cultivars (**Figures 9A-F**). However, at 30°C, *StWRKY75*-overexpressing lines in both cultivars exhibited significantly enhanced photosynthetic rate (**Figures 9A, B**), transpiration rate (**Figures 9C, D**), and stomatal conductance (**Figures 9E, F**) compared to NT plants. Conversely, *StWRKY75*-knockdown lines showed reduced photosynthetic performance. Under severe heat stress (35°C), growth inhibition was observed in all potato plants due to thermal damage. Nevertheless, *StWRKY75*-overexpressing lines maintained significantly higher photosynthetic rate (**Figures 9A, B**), transpiration rate (**Figures 9C, D**), and stomatal conductance (**Figures 9E, F**) compared to NT controls in both cultivars. In contrast, *StWRKY75*-knockdown plants displayed markedly reduced gas exchange parameters relative to NT lines, demonstrating the crucial regulatory role of *StWRKY75* in maintaining photosynthetic performance under heat stress.

## 4 Discussion

Several WRKY TFs have been extensively studied in plants under heat stress conditions (Ding et al., 2021; Yang et al., 2022; Liu et al., 2024; Yang et al., 2025). However, there is a lack of comprehensive research on how WRKY TFs regulate heat stress responses in potatoes. The sequence alignment demonstrated that StWRKY75 harbors the highly conserved WRKYGQK domain at the N-terminus, followed by a C_2_H_2_ zinc-finger motif (CX_5_-C-X_23_-H-X_1_-H), a characteristic feature of Group II WRKY TFs (**Figure 1A**). Phylogenetic analysis classified the examined plant species into different clusters, with members within the same cluster exhibiting sequence homology and functional conservation (**Figure 1B**). These findings align with prior research on ZmWRKY106, which shares a 28.47% mean sequence identity with its orthologs in rice (*Oryza sativa*), *Arabidopsis* (*Arabidopsis thaliana*), and barley (*Hordeum vulgare*). Like StWRKY75, ZmWRKY106 possesses the conserved WRKYGQK domain and C_2_H_2_ zinc-finger motif, further supporting its classification within Group II. However, sequences outside these conserved regions exhibited significant divergence. Phylogenetic analysis indicated that ZmWRKY106 is most closely related to OsWRKY13 (61% bootstrap support) and HvWRKY39 (100% bootstrap support), suggesting evolutionary and functional conservation among these orthologs (Wang et al., 2018). Furthermore, through differential expression analysis under various heat stress conditions (**Figures 3**, **4**), we confirmed that *StWRKY75* was significantly induced under elevated temperatures (30°C and 35°C), suggesting its crucial functional role in heat stress response. To further understand the response of WRKY TFs to heat stress, the expression patterns of *AtWRKY25*, *AtWRKY26*, and *AtWRKY33* were analyzed in *Arabidopsis thaliana* using qRT-PCR following heat stress treatments at 42, 45, and 48°C. The results demonstrated that *AtWRKY25* and *AtWRKY26* were induced at 45°C and 48°C, with expression profiles similar to those observed at 42°C. However, the fold-induction of *AtWRKY26* in the wild type was consistently lower than that of *AtWRKY25* at each time point following exposure to different high temperatures. In contrast, the expression of *AtWRKY33* steadily decreased under heat stress, highlighting the distinct regulatory responses of these WRKY TFs to high temperatures (Li et al., 2011). Earlier investigations revealed differential expression patterns of *BcWRKY22* in various Chinese cabbage tissues (roots, stems, leaves) when exposed to heat stress. The results revealed that *BcWRKY22* exhibited higher expression levels in roots and stems compared to leaves, with the highest expression observed in roots under heat stress. The qRT-PCR results indicated a prompt increase in *BcWRKY22* mRNA accumulation in foliar tissues 1 hour and in roots 3 hours after heat shock (HS) induction. Notably, high expression levels were maintained in both tissues even 24 hours after HS, compared to the control condition at 24°C (Wang et al., 2023). Consistent with these findings, our study demonstrated a similar research trend with different heat ingredients (30°C and 35°C), where the highest expression levels of *StWRKY75* were detected in leaves of cultivars ‘*Atlantic*’ and ‘*Desiree*’ at 48 hours after heat stress treatments, as shown in **Figures 2** and **3C, D**.

Prior studies demonstrated that WRKY TFs mostly possess nuclear localization signals (NLS), enabling their translocation into the nucleus, and interact with DNA to regulate gene expression (Jiang et al., 2017). To further confirm the subcellular localization of WRKY proteins, a PEG-mediated transient expression system was employed in *Zea mays* mesophyll protoplasts using the vector p16318h-ZmWRKY106. Confocal laser scanning microscopy revealed that the p16318h-ZmWRKY106 fusion protein was predominantly localized in the nucleus after an 18-hour incubation in the dark, whereas the control vector (EGFP) exhibited fluorescence in the membrane, cytoplasm, and nucleus (Wang et al., 2018). Similarly, in this study, the subcellular localization of StWRKY75 in potato was investigated under heat stress. Green fluorescent protein (GFP) signals indicated that the StWRKY75 transcription factor was localized to the nucleus, consistent with its role as a transcriptional regulator. In contrast, the control vector (GFP) displayed fluorescence in the membrane, cytoplasm, and nucleus (**Figure 4**). Growth indicators, including plant height, tuber weights, shoot and root fresh and dry weights, leaf number, leaf area, root length, and total biomass, are critical parameters for evaluating the impact of abiotic stress and understanding plant adaptive mechanisms under unfavorable environmental conditions (Pandey et al., 2017). In the present study, under heat stress, *StWRKY75*-overexpression lines demonstrated enhanced growth across multiple indices, including plant height, tuber weight per plant, total fresh and dry weights, as well as root fresh and dry weights, relative to the NT potato lines. Conversely, RNAi-mediated gene silencing resulted in the opposite trend, as shown in **Table 1**. These findings align with previous research, which demonstrated that overexpression of *AtWRKY30* in wheat enhances growth and biomass accumulation under heat and drought stresses. Specifically, under combined heat and drought conditions, both transformed and wild-type plants exhibited reductions in growth indicators, such as shoot fresh weight, shoot length, root fresh weight, and root length, compared to optimal growth conditions. However, *AtWRKY30-*overexpressing wheat plants exhibited significant improvements in growth and biomass parameters relative to wild-type plants under these stresses, highlighting the role of *AtWRKY30* in enhancing stress resilience and promoting root and shoot development (El-Esawi et al., 2019).

Plants have developed sophisticated biochemical defense systems involving antioxidant enzymes (APX, SOD, CAT, POD) to combat environmental stresses. The activity levels of these enzymes, together with oxidative markers including MDA and H_2_O_2_, serve as dependable biomarkers of plant stress responses. Increased enzymatic activity of APX, SOD, CAT, and POD coupled with decreased accumulation of MDA and H_2_O_2_ demonstrates enhanced oxidative defense mechanisms and greater stress adaptation capacity (Rajput et al., 2021). Under harsh environmental conditions, plant cells exhibit marked elevation in ROS production, particularly O_2_^-^, H_2_O_2_, and HO^-^. The potent oxidative capacity of these molecules facilitates deleterious interactions with essential cellular constituents, resulting in the degradation of lipids, alteration of protein structures, and nucleic acid lesions that collectively compromise cell viability (García-Caparrós et al., 2021). As an important osmolyte, proline contributes significantly to ROS detoxification and improves plant resilience against multiple abiotic stressors (Hayat et al., 2012). Results from this investigation confirmed the functional role of the *StWRKY75* TF in modulating oxidative stress defense mechanisms across two commercially significant potato cultivars (*Solanum tuberosum* L. cultivars; ‘*Atlantic*’ and *‘Desiree’*) under differential heat stress treatments (20°C as the control and 30°C/35°C as heat stress treatments). Under control conditions, all tested plants (transgenic and NT) remained unchanged. Under varying heat stress conditions, *StWRKY75*-overexpressing plants exhibited enhanced activities of APX (**Figures 5**, **6A**), CAT (**Figures 5**, **6B**), POD (**Figures 5**, **6C**), and SOD (**Figures 5**, **6D**), as well as increased proline content (**Figures 5**, **6E**), compared to non-transformed (NT) and RNA interference (RNAi) plants showed opposite outcomes. Additionally, *StWRKY75-*overexpressing plants displayed significantly reduced levels of MDA (**Figures 5**, **6F**) under heat stress, indicating lower oxidative damage. In contrast, RNAi plants exhibited increased oxidative damage due to the downregulation of *StWRKY75*. The experimental data validate prior research, which demonstrated that overexpression of *ZmWRKY106* in maize enhanced antioxidant enzyme activities (SOD, POD, and CAT) and reduced ROS levels under drought and heat stress, thereby improving abiotic tolerance (Wang et al., 2018). While the current study focused on heat stress (30°C and 35°C) rather than drought stress, the results align with earlier findings, highlighting the conserved role of WRKY TFs in enhancing antioxidant defense mechanisms under abiotic stress.

Furthermore, we analyzed the expression of stress-responsive genes, including *StAPX*, *StSOD*, *StCAT*, *StPOD*, *StP5SC*, *StHSP20*, *StHSP70*, and *StHSP90* in two potato cultivars, ‘*Atlantic*’ and *‘Desiree’*, *StWRKY75*-overexpressing and RNAi plants under varying degrees of heat stress treatments (30°C and 35°C). No differential expression of stress-related genes was observed under control conditions (20°C) (**Figures 7**, **8**). The relative mRNA expression analysis revealed significant upregulation of StAPX (**Figures 7**, **8A**), *StCAT* (**Figures 7**, **8C**), *StSOD* (**Figures 7**, **8C**), *StPOD* (**Figures 7**, **8D**), *StP5SC* (**Figures 7**, **8E**), *StHSP20* (**Figures 7**, **8F**), *StHSP70* and (**Figures 7**, **8G**), *StHSP90* (**Figures 7**, **8H**) in *StWRKY75-*overexpressing plants, while these genes were downregulated in RNAi plants after heat stress treatments. These results suggest that *StWRKY75* plays a pivotal role in modulating the transcriptional cascade of stress-responsive genes, thereby enhancing heat tolerance in potato plants. Similar trends were observed in a previous study on drought stress in *Arabidopsis*, where overexpression of *TaWRKY31* upregulated genes involved in osmotic adjustment and ROS scavenging, including *AtNCED3, AtABA2, AtSnRK2.2, AtABI1, AtABF3, AtP5CS1, AtSOD(Cu/Zn), AtPOD, AtCAT, AtRD29A*, and *AtRD29B* (Ge et al., 2024). Our study only analyzed the antioxidant-related and proline biosynthesis-related stress-responsive genes under heat stress. These data collectively underscore how WRKY TFs contribute to stress tolerance by optimizing ROS-scavenging enzymes and regulating key stress-associated genes. Heat shock proteins (HSPs) are evolutionarily conserved molecular chaperones that enhance stress adaptation by maintaining protein homeostasis under abiotic stresses, including heat, drought, and salinity (Wang et al., 2022). The current research showed an upregulation in heat shock protein-related genes, including *StHSP20* (**Figures 7**, **8F**), *StHSP70* (**Figures 7**, **8G**), and *StHSP90* (**Figures 7**, **8H**) in *StWRKY75*-overexpressing lines, which showed consistency with a previous report. The overexpression of *PtWRKY2* significantly reduced ROS accumulation through enhanced enzymatic activity of key antioxidant enzymes related genes, including catalase-1 (*CAT1*), superoxide dismutase-1 (*SOD1*), and peroxidase-34 (*POD34*). Additionally, transcript levels of heat shock-associated genes (*HSP70*, *HSP17.4*) were markedly elevated in transgenic *Arabidopsis* lines (Liu et al., 2024). These findings demonstrate that *PtWRKY2* orchestrates a coordinated upregulation of thermotolerance-related genes, enhancing both protein homeostasis and oxidative stress mitigation under heat stress conditions. Another study described that the *WRKY25* knockdown slightly increased HSP20 expression, whereas *WRKY33* knockdown markedly enhanced it in cotton (Ehsan et al., 2023).

Heat stress has a significant impact on photosynthesis, which is a critical physiological process in plants. The influence of high temperatures on photosynthesis parameters (Pn, Gs, and E) can vary depending on the intensity and duration of the heat, as well as the plant species and their ability to tolerate high temperatures. Additionally, heat stress negatively impacts photosynthesis by reducing the efficiency of light capture, impairing carbon fixation, increasing photorespiration, and causing oxidative damage (Zahra et al., 2023). Our investigation demonstrated that under varying degrees of heat stress conditions (20°C, 30°C, and 35°C), transgenic plants overexpressing *StWRKY75* exhibited significantly enhanced photosynthetic rate (**Figures 9A, B**), transpiration rate (**Figures 9C, D**), and stomatal conductance (**Figures 9E, F**) compared to the NT control plants. Conversely, plants with *StWRKY75* knockdown expression displayed the opposite phenotypic trends. These findings provide evidence for the regulatory role of *StWRKY75* in modulating physiological responses under heat stress, thereby confirming its transcriptional regulatory function. In peanut (*Arachis hypogaea*), gas exchange parameters (Pn, Gs, and E) were significantly elevated in *AhWRKY75* transgenic lines compared to NT control plants under salt stress conditions. Conversely, the intercellular CO_2_ concentration (Ci) was significantly lower in the transgenic plants relative to the controls (Zhu et al., 2021). Our current investigation demonstrated a comparable trend concerning Pn, Gs, and E; however, the key distinction lies in the application of different abiotic stress conditions. This suggests that *StWRKY75* overexpression may confer enhanced photosynthetic performance and stomatal regulation across diverse stress environments, justifying further exploration into its role in plant stress tolerance mechanisms.

Although the critical role of *StWRKY75* in potato heat stress response has been validated through transgenic approaches in this study, several limitations remain to be addressed in future research: Potential functional redundancy among WRKY transcription factor family members: Other homologous genes may partially compensate for *StWRKY75* deficiency, possibly resulting in less pronounced phenotypic changes in RNAi lines. Future studies should employ multiplex gene knockout (CRISPR-Cas9 genome editing) or protein interaction analyses (such as yeast two-hybrid, BIFC, and Co-IP) to verify gene interaction networks. Lack of field validation: Controlled laboratory conditions (e.g., constant temperature, artificial lighting) cannot fully simulate complex field stress conditions. Subsequent multi-year and multi-location field trials under natural conditions, combined with climate data analysis, are required to evaluate the agronomic value of this gene. While *StWRKY75* was identified as a central regulator of antioxidant enzymes and HSPs in thermotolerance, its direct target genes remain unverified. Additional experiments, including chromatin immunoprecipitation sequencing (ChIP-seq), yeast one-hybrid assay, and electrophoretic mobility shift assay (EMSA), should be conducted to identify DNA binding sites and further elucidate the downstream molecular mechanisms of heat tolerance. Sampling only at 48 hours after stress treatment may miss early responses (such as ROS burst within 0–48 hours) and long-term adaptation to heat stress. Additional sampling time points should be included to avoid insufficient dynamic monitoring of relevant physiological indicators.

## 5 Conclusion

This study demonstrates that StWRKY75, a nuclear-localized Group II WRKY transcription factor, plays a pivotal role in potato thermotolerance. Overexpression of *StWRKY75* significantly enhances thermotolerance by elevating the activities of antioxidant enzymes (APX, CAT, POD, SOD), increasing proline accumulation, optimizing photosynthetic parameters (photosynthesis, transpiration, stomatal conductance), and reducing MDA accumulation. Concurrently, *StWRKY75* upregulates key stress-responsive genes, including *StAPX*, *StCAT*, *StPOD*, *StSOD*, *StP5CS*, and heat shock protein coding genes (*StHSP20*, *StHSP70*, *StHSP90*). Conversely, *StWRKY75*-knockdown plants exhibit compromised thermotolerance with opposing physiological trends. These findings highlight *StWRKY75* as a strategic target for molecular breeding to improve heat resilience in potato and related crops, offering a sustainable solution to mitigate climate change impacts on agricultural productivity.

## Data Availability

The datasets used and/or analyzed during the current study are available from the corresponding author upon reasonable request.

## References

[B1] Adekanmbi T. Wang X. Basheer S. Liu S. Yang A. Cheng H. (2024). Climate change impacts on global potato yields: a review. Environ. Res. Clim. 3, 012001. doi: 10.1088/2752-5295/ad0e13

[B2] Aebi H. (1984). “Catalase *in Vitro* ,” in Methods in Enzymology, vol. 105 . Ed. Packer L. (Academic Press, San Diego, California), 121–126. doi: 10.1016/s0076-6879(84)05016-3 6727660

[B3] Ahn Y. J. Claussen K. Zimmerman J. L. (2004). Genotypic differences in the heat-shock response and thermotolerance in four potato cultivars. Plant Sci. 166, 901–911. doi: 10.1016/j.plantsci.2003.11.027

[B4] Aien A. Chaturvedi A. K. Bahuguna R. N. Pal M. (2017). Phenological sensitivity to high-temperature stress determines dry matter partitioning and yield in potato. Indian J. Plant Physiol. 22, 63–69. doi: 10.1007/s40502-016-0270-z

[B5] Basu P. S. Minhas J. S. (1991). Heat tolerance and assimilate transport in different potato genotypes. J. Exp. Bot. 42, 861–866. doi: 10.1093/jxb/42.7.861

[B6] Bates L. S. Waldren R. P. Teare I. D. (1973). Rapid determination of free proline for water-stress studies. Plant Soil 39, 205–207. doi: 10.1007/BF00018060

[B7] Bender F. D. Sentelhas P. C. (2020). Assessment of regional climate change impacts on Brazilian potato tuber yield. Int. J. Plant Prod. 14, 647–661. doi: 10.1007/s42106-020-00111-7

[B8] Cheong Y. H. Chang H. S. Gupta R. Wang X. Zhu T. Luan S. (2002). Transcriptional profiling reveals novel interactions between wounding, pathogen, abiotic stress, and hormonal responses in *Arabidopsis* . Plant Physiol. 129, 661–677. doi: 10.1104/pp.002857 12068110 PMC161692

[B9] Ciolkowski I. Wanke D. Birkenbihl R. P. Somssich I. E. (2008). Studies on DNA-binding selectivity of WRKY transcription factors lend structural clues to WRKY-domain function. Plant Mol. Boil 68, 81–92. doi: 10.1007/s11103-008-9353-1 PMC2493524 18523729

[B10] Dereeper A. Audic S. Claverie J. M. Blanc G. (2010). Blast-explorer helps you build datasets for phylogenetic analysis. BMC Evol. Biol. 10, 8. doi: 10.1186/1471-2148-10-8 20067610 PMC2821324

[B11] Devaux A. Goffart J. P. Petsakos A. Kromann P. Gatto M. Okello J.. (2020). Global food security, contributions from sustainable potato agri-food systems. potato crop: Its agricult Nutr. Soc. contrib to humankind 3–35. doi: 10.1007/978-3-030-28683-5

[B12] Ding L. Wu Z. Teng R. Xu S. Cao X. Yuan G.. (2021). *LlWRKY39* is involved in thermotolerance by activating *LlMBF1c* and interacting with *LlCaM3* in lily (*Lilium longiflorum*). Hortic. Res. 8. doi: 10.1038/s41438-021-00473-7 PMC7862462 33542226

[B13] Dong Q. Zheng W. Duan D. Huang D. Wang Q. Liu C.. (2020). *MdWRKY30*, a group IIa WRKY gene from apple, confers tolerance to salinity and osmotic stresses in transgenic apple callus and *Arabidopsis* seedlings. Plant Sci. 299, 110611. doi: 10.1016/j.plantsci.2020.110611 32900448

[B14] Duan D. Yi R. Ma Y. Dong Q. Mao K. Ma F. (2023). Apple WRKY transcription factor *MdWRKY56* positively modulates drought stress tolerance. Environ. Exp. Bot. 212, 105400. doi: 10.1016/j.envexpbot.2023.105400

[B15] Ehsan A. Naqvi R. Z. Azhar M. Awan M. J. A. Amin I. Mansoor S.. (2023). Genome-wide analysis of the WRKY gene family and negative regulation of *GhWRKY25* and *GhWRKY33* reveal their role in whitefly and drought stress tolerance in cotton. Genes 14, 171. doi: 10.3390/genes14010171 36672912 PMC9859137

[B16] El-Esawi M. A. Al-Ghamdi A. A. Ali H. M. Ahmad M. (2019). Overexpression of *AtWRKY30* transcription factor enhances heat and drought stress tolerance in wheat (Tri*ticum aestivum* L.). Genes 10, 163. doi: 10.3390/genes10020163 30791662 PMC6410048

[B17] Eulgem T. Rushton P. J. Robatzek S. Somssich I. E. (2000). The WRKY superfamily of plant transcription factors. Trends Plant Sci. 5, 199–206. doi: 10.1016/S1360-1385(00)01600-9 10785665

[B18] García-Caparrós P. De Filippis L. Gul A. Hasanuzzaman M. Ozturk M. Altay V.. (2021). Oxidative stress and antioxidant metabolism under adverse environmental conditions: a review. Bot. Rev. 87, 421–466. doi: 10.1007/s12229-020-09231-1

[B19] Gautam S. Scheuring D. C. Koym J. W. Vales M. I. (2024). Assessing heat tolerance in potatoes: Responses to stressful Texas field locations and controlled contrasting greenhouse conditions. Front. Plant Sci. 15. doi: 10.3389/fpls.2024.1364244 PMC11128680 38803598

[B20] Gautam S. Solis-Gracia N. Teale M. K. Mandadi K. Silva J. A. D. Vales M. I. (2021). Development of an *in vitro* microtuberization and temporary immersion bioreactor system to evaluate potato heat stress tolerance (Solanum tuberosum L.). Front. Plant Sci. 12. doi: 10.3389/fpls.2021.700328 PMC8385365 34456944

[B21] Ge M. Tang Y. Guan Y. Lv M. Zhou C. Ma H.. (2024). *TaWRKY31*, a novel WRKY transcription factor in wheat, regulates plant drought stress tolerance. BMC Plant Biol. 24, 27. doi: 10.1186/s12870-023-04709-7 38172667 PMC10763432

[B22] Giannopolitis C. N. Ries S. K. (1977). Superoxide dismutase: I. Occurrence in higher plants. Plant Physiol. 59, 309–314. doi: 10.1104/pp.59.2.309 16659839 PMC542387

[B23] Gonzalez-Jimenez J. Andersson B. Wiik L. Zhan J. (2023). Modelling potato yield losses caused by *Phytophthora infestans:* Aspects of disease growth rate, infection time and temperature under climate change. Field Crops Res. 299, 108977. doi: 10.1016/j.fcr.2023.108977

[B24] Guillemette A. M. Hernández Casanova G. Hamilton J. P. Pokorná E. Dobrev P. I. Motyka V.. (2025). The physiological and molecular responses of potato tuberization to projected future elevated temperatures. Plant Physiol. 197, 664. doi: 10.1093/plphys/kiae664 PMC11683837 39688842

[B25] Hayat S. Hayat Q. AlYemeni M. N. Wani A. S. Pichtel J. Ahmad A. (2012). Role of proline under changing environments: a review. Plant Signal Behav. 7, 1456–1466. doi: 10.4161/psb.21949 22951402 PMC3548871

[B26] Heath R. L. Packer L. (1968). Photo-oxidation in isolated chloroplasts. I. Kinetics and stoichiometry of fatty acid peroxidation. Arch. Biochem. Biophys. 125, 189–198. doi: 10.1016/0003-9861(68)90654-1 5655425

[B27] Hichri I. Muhovski Y. Žižková E. Dobrev P. I. Gharbi E. Franco-Zorrilla J. M.. (2017). The *Solanum lycopersicum* WRKY3 transcription factor (*SlWRKY3*) is involved in salt stress tolerance in tomato. Front. Plant Sci. 8, 1343. doi: 10.3389/fpls.2017.01343 28824679 PMC5534461

[B28] Ishiguro S. Nakamura K. (1994). Characterization of a cDNA encoding a novel DNA-binding protein, *SPF1*, that recognizes SP8 sequences in the 5′ upstream regions of genes coding for sporamin and β-amylase from sweet potato. Mol. Gen. Genet. 244, 563–571. doi: 10.1007/BF00282746 7969025

[B29] Jiang Y. Guo L. Ma X. Zhao X. Jiao B. Li C.. (2017). The WRKY transcription factors *PtrWRKY18* and *PtrWRKY35* promote Melampsora resistance in Populus. Tree Physiol. 37, 665–675. doi: 10.1093/treephys/tpx008 28338710

[B30] Kumar Y. Singh R. Kumar A. (2021). Performance of SUBSTOR model on growth and yield of potato varieties under different planting dates in a sub-tropical environment. J. Agrometeorol 23, 213–220. doi: 10.54386/jam.v23i2.71

[B31] Li G. Cao C. Yang H. Wang J. Wei W. Zhu D.. (2020). Molecular cloning and potential role of DiSOC1s in flowering regulation in Davidia involucrata Baill. Plant Physiol. Biochem. 157, 453–459. doi: 10.1016/j.plaphy.2020.11.003 33218844

[B32] Li S. Fu Q. Chen L. Huang W. Yu D. (2011). Arabidopsis thaliana WRKY25, WRKY26, and *WRKY33* coordinate the induction of plant thermotolerance. Planta 233, 1237–1252. doi: 10.1007/s00425-011-1375-2 21336597

[B33] Liu D. Cui W. Bo C. Wang R. Zhu Y. Duan Y.. (2024). *PtWRKY2*, a WRKY transcription factor from *Pinellia ternata* confers heat tolerance in *Arabidopsis* . Sci. Rep. 14, 13807. doi: 10.1038/s41598-024-64560-0 38877055 PMC11178784

[B34] Livak K. J. Schmittgen T. D. (2001). Analysis of relative gene expression data using real-time quantitative PCR and the 2^–ΔΔCT^ method. Methods 25, 402–408. doi: 10.1006/meth.2001.1262 11846609

[B35] Lu H. Klocko A. L. Brunner A. M. Ma C. Magnuson A. C. Howe G. T.. (2019). RNA Interference suppression of AGAMOUS and SEEDSTICK alters floral organ identity and impairs floral organ determinacy, ovule differentiation, and seed hair development in *Populus* . New Phytol. 222, 923–937. doi: 10.1111/nph.15648 30565259 PMC6590139

[B36] Maehly A. C. Chance B. (1954). “The assay of catalases and peroxidases,” in Methods of biochemical analysis, vol. 1 . Ed. Glick D. (Interscience Publishers, New York, USA), 357–424. doi: 10.1002/9780470110171.ch14 13193536

[B37] Nakano Y. Asada K. (1981). Hydrogen peroxide is scavenged by ascorbate-specific peroxidase in spinach chloroplasts. Plant Cell Physiol. 22, 867–880. doi: 10.1093/oxfordjournals.pcp.a076232

[B38] Pandey P. Irulappan V. Bagavathiannan M. V. Senthil-Kumar M. (2017). Impact of combined abiotic and biotic stresses on plant growth and avenues for crop improvement by exploiting physio-morphological traits. Front. Plant Sci. 8. doi: 10.3389/fpls.2017.00537 PMC5394115 28458674

[B39] Pandey S. P. Somssich I. E. (2009). The role of WRKY transcription factors in plant immunity. Plant Physiol. 150, 1648–1655. doi: 10.1104/pp.109.138990 19420325 PMC2719123

[B40] Phukan U. J. Jeena G. S. Shukla R. K. (2016). WRKY transcription factors: molecular regulation and stress responses in plants. Front. Plant Sci. 7. doi: 10.3389/fpls.2016.00760 PMC4891567 27375634

[B41] Rajput V. D. Harish Singh R. K. Verma K. K. Sharma L. Quiroz-Figueroa F. R.. (2021). Recent developments in the enzymatic antioxidant defense mechanism in plants with special reference to abiotic stress. Biology 10, 267. doi: 10.3390/biology10040267 33810535 PMC8066271

[B42] Rolando J. L. Ramírez D. A. Yactayo W. Monneveux P. Quiroz R. (2015). Leaf greenness as a drought tolerance-related trait in potato (*Solanum tuberosum* L.). Environ. Exp. Bot. 110, 27–35. doi: 10.1016/j.envexpbot.2014.09.006

[B43] Rykaczewska K. (2015). The effect of high temperature occurring in subsequent stages of plant development on potato yield and tuber physiological defects. Am. J. Potato Res. 92, 339–349. doi: 10.1007/s12230-015-9436-x

[B44] Singh B. Kukreja S. Goutam U. (2020). Impact of heat stress on potato (*Solanum tuberosum* L.): Present scenario and future opportunities. J. Hortic. Sci. Biotech 95, 407–424. doi: 10.1080/14620316.2019.1700173

[B45] Song Y. Chen L. Zhang L. Yu D. (2010). Overexpression of the *OsWRKY72* gene interferes with the abscisic acid signal and auxin transport pathway of. Arabidop J. Biosci. 35, 459–471. doi: 10.1007/s12038-010-0051-1 20826955

[B46] Sparkes I. A. Runions J. Kearns A. Hawes C. (2006). Rapid, transient expression of fluorescent fusion proteins in tobacco plants and generation of stably transformed plants. Nat. Protoc. 1, 2019–2025. doi: 10.1038/nprot.2006.286 17487191

[B47] Stark J. C. Love S. L. King B. A. Marshall J. M. Bohl W. H. Salaiz T. (2013). Potato cultivar response to seasonal drought patterns. Am. J. Potato Res. 90, 207–216. doi: 10.1007/s12230-012-9285-9

[B48] Thompson J. D. Higgins D. G. Gibson T. J. (1994). CLUSTAL W: improving the sensitivity of progressive multiple sequence alignment through sequence weighting, position-specific gap penalties, and weight matrix choice. Nucl. Acids Res. 22, 4673–4680. doi: 10.1093/nar/22.22.4673 7984417 PMC308517

[B49] Wang H. Dong Z. Chen J. Wang M. Ding Y. Xue Q.. (2022). Genome-wide identification and expression analysis of the Hsp20, Hsp70, and Hsp90 gene family in *Dendrobium officinale* . Front. Plant Sci. 13. doi: 10.3389/fpls.2022.979801 PMC9399769 36035705

[B50] Wang Y. Gai W. Yuan L. Shang L. Li F. Gong Z.. (2024). Heat-inducible *SlWRKY3* confers thermotolerance by activating the *SlGRXS1* gene cluster in tomato. Hortic. Plant J. 10, 515–531. doi: 10.1016/j.hpj.2022.12.006

[B51] Wang H. Gao Z. Chen X. Li E. Li Y. Zhang C.. (2023). *BcWRKY22* activates *BcCAT2* to enhance catalase (CAT) activity and reduce hydrogen peroxide (H_2_O_2_) accumulation, promoting thermotolerance in non-heading Chinese cabbage (*Brassica campestris* ssp. chinensis). Antioxidants 12, 1710. doi: 10.3390/antiox12091710 37760013 PMC10525746

[B52] Wang C. T. Ru J. N. Liu Y. W. Li M. Zhao D. Yang J. F.. (2018). Maize WRKY transcription factor *ZmWRKY106* confers drought and heat tolerance in transgenic plants. Int. J. Mol. Sci. 19, 3046. doi: 10.3390/ijms19103046 30301220 PMC6213049

[B53] Wani S. H. Anand S. Singh B. Bohra A. Joshi R. (2021). WRKY transcription factors and plant defense responses: latest discoveries and future prospects. Plant Cell Rep. 40, 1071–1085. doi: 10.1007/s00299-021-02691-8 33860345

[B54] Wu X. Shiroto Y. Kishitani S. Ito Y. Toriyama K. (2009). Enhanced heat and drought tolerance in transgenic rice seedlings overexpressing *OsWRKY11* under the control of the *HSP101* promoter. Plant Cell Rep. 28, 21–30. doi: 10.1007/s00299-008-0614-x 18818929

[B55] Yang S. Cai W. Shen L. Cao J. Liu C. Hu J.. (2022). A *CaCDPK29–CaWRKY27b* module promotes *CaWRKY40-*mediated thermotolerance and immunity to *Ralstonia solanacearum* in pepper. New Phytol. 233, 1843–1863. doi: 10.1111/nph.17891 34854082

[B56] Yang L. Fang S. Liu L. Zhao L. Chen W. Li X.. (2025). WRKY transcription factors: Hubs for regulating plant growth and stress responses. J. Integr. Plant Biol. 67, 488–509. doi: 10.1111/jipb.13828 39815727

[B57] Yu Y. Wu Y. He L. (2023). A wheat WRKY transcription factor *TaWRKY17* enhances tolerance to salt stress in transgenic *Arabidopsis* and wheat plants. Plant Mol. Biol. 113, 171–191. doi: 10.1007/s11103-023-01381-1 37902906

[B58] Zahra N. Hafeez M. B. Ghaffar A. Kausar A. Al Zeidi M. Siddique K. H.. (2023). Plant photosynthesis under heat stress: Effects and management. Environ. Exp. Bot. 206, 105178. doi: 10.1016/j.envexpbot.2022.105178

[B59] Zhang C. Wang D. Yang C. Kong N. Shi Z. Zhao P.. (2017). Genome-wide identification of the potato WRKY transcription factor family. PloS One 12, e0181573. doi: 10.1371/journal.pone.0181573 28727761 PMC5519183

[B60] Zhu H. Jiang Y. Guo Y. Huang J. Zhou M. Tang Y.. (2021). A novel salt-inducible WRKY transcription factor gene, *AhWRKY75*, confers salt tolerance in transgenic peanut. Plant Physiol. Biochem. 160, 175–183. doi: 10.1016/j.plaphy.2021.01.014 33497848

[B61] Zhu X. Li W. Zhang N. Jin H. Duan H. Chen Z.. (2024). *StMAPKK5* responds to heat stress by regulating potato growth, photosynthesis, and antioxidant defenses. Front. Plant Sci. 15. doi: 10.3389/fpls.2024.1392425 PMC11137293 38817936

