# Supplementary material for: Functional analysis of StWRKY75 gene in response to heat stress in potato (Solanum tuberosum L.)
Source: Front Plant Sci. 2025 Jun 30;16:1617625. doi: 10.3389/fpls.2025.1617625 (PMC12256211; doi:10.3389/fpls.2025.1617625)
Supplement: Supplementary file 1 [file DataSheet1.pdf]

Title: Functional Analysis of *StWRKY75* Gene in Response to Heat Stress in Potato (*Solanum tuberosum* L.)

Supplementary Materials

1. Supplementary Figures

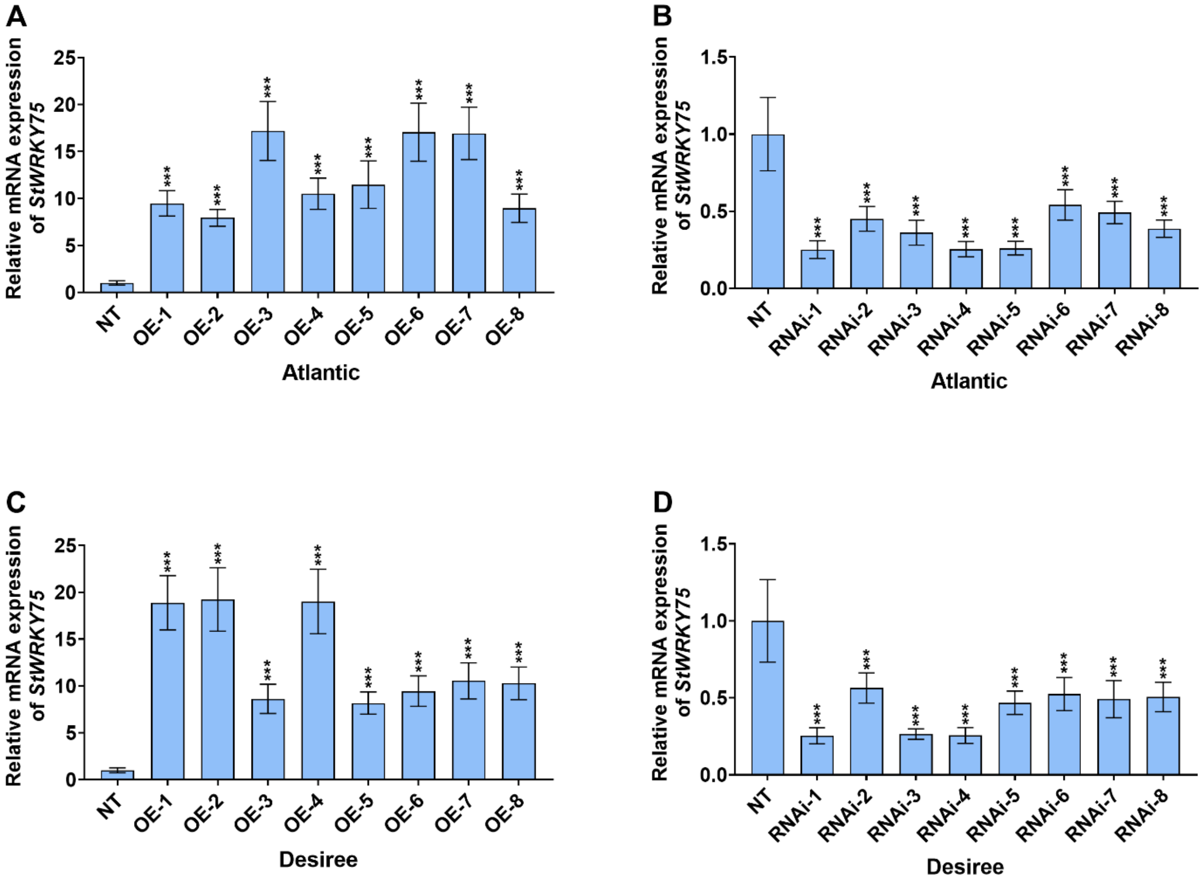

Figure S1. The relative mRNA expression of *StWRKY75* in Potato. In the '*Atlantic*' cultivar: NT: non-transgenic plants; OE, pBI121-EGFP-StWRKY75-transgenic plants (OE-1, OE-2, OE-3, OE-4, OE-5, OE-6, OE-7, and OE-8); RNAi, pART-StWRKY75-RNAi-transgenic plants (RNAi-1, RNAi-2, RNAi-3, RNAi-4, RNAi-5, RNAi-6, RNAi-7 and RNAi-8). In the '*Desiree*' cultivar: NT: non-transgenic plants; OE, pBI121-EGFP-StWRKY75-transgenic plants (OE-1, OE-2, OE-3, OE-4, OE-5, OE-6, OE-7, and OE-8); RNAi, pART-StWRKY75-RNAi-transgenic plants (RNAi-1, RNAi-2, RNAi-3, RNAi-4, RNAi-5, RNAi-6, RNAi-7, and RNAi-8). The data are presented as mean ± standard deviation. P-values (***$P < 0.001$) were calculated through ordinary two-way ANOVA followed by Tukey's multiple comparisons test with a sample size of n = 9.

2. Supplementary Tables

Table S1. Protein IDs of the *WRKY75* transcription factor in different plant species.

Plant species	Protein	Protein ID

Arabidopsis thaliana	AtWRKY75	NP_196812.1
Solanum tuberosum	StWRKY75	NP_001275604.1
Solanum lycopersicum	SlWRKY75	NP_001310244.1
Physalis pubescens	PbWRKY75	AZB50357.1
Capsicum annuum	CaWRKY75	XP_016555165.1
Nicotiana tomentosiformis	NtWRKY75	XP_009615508.1
Prunus mume	PmWRKY75	XP_008222197.1
Mangifera indica	MiWRKY75	XP_044488111.1 w
Lycium barbarum	LbWRKY75	XP_060214229.1
Nicotiana sylvestris	NsWRKY75	XP_009767666.1
Pyrus communis	PcWRKY75	XP_068333417.1
Camellia lanceoleosa	ClWRKY75	KAI7998167.1
Argentina anserine	AaWRKY75	XP_050372951.1
Malus domestica	MdWRKY75	XP_008389898.1
Ipomoea batatas	IbWRKY75	GMD51004.1
Rosa chinensis	RcWRKY75	XP_024197588.1
Gossypium raimondii	GrWRKY75	XP_012446088.1
Camellia sinensis	CsWRKY75	XP_028124928.1
Hibiscus syriacus	HsWRKY75	XP_039069607.1

The above-mentioned proteins can be directly accessed through the National Center for Biotechnology Information (NCBI database) (https://www.ncbi.nlm.nih.gov/guide/).

Table S2. Sequences of primers used in the present study.

**Gene ID**	**Gene**	**Forward (5'-3')**	**Reverse (5'-3')**
**qRT-PCR:**			
XM_006347752.2	*StEf1α*	GGTTGTATCTCTTCCGATAAAGGC	GGTTGTATCTCTTCCGATAAAGGC
AB041343	*StAPX*	CTCCTCTGTGATCCTGCTTTC	GAGAGTGTCAAGTGAGCCTTAG
AY442179	*StCAT*	CCATGCTGAGGTGTATCCTATTC	CCTTTCTCCTGGTTGCTTGA
AF354748	*StSOD*	CATTGGAAGAGCTGTTGTTGTT	ATCCTTCCGCCAGCATTT
XM_006362636.2	*StPOD*	AGATGTTGTGGCCATGTCTGG	GCTTGTGTTGAAGGATGGAGC
XM_015308529.1	*StP5CS*	TGCAATGCAATGGAAACGCT	ACAATTTCCACGGTGCAAGC
JX576239	*StHSP20*	GGAGAGAGGAATGTGGAGAAAG	CGCATTCTCCGGAAGTCTAAA
Z11982.1	*StHSP70*	GTGTTGGTGTATGGCAAAACGA	AGCAACTTGATTCTTGGCTGC
HS106768.1	*StHSP90*	CAGTGGTATCAACGCAGAGTAA	TCCTTCACAGACTTGTCATTCTT
NM_001288675.1	*StWRKY75*	CCAAACAAGGAGCCAAGTTGA	GCATCGATAATAGCTCCTTGGGA

The above-mentioned genes can be directly accessed through the National Center for Biotechnology Information (NCBI database) (https://www.ncbi.nlm.nih.gov/guide/).

3. Supplementary data 1

3.1 Assessment of physiological indicators

The following physiological indicators were measured as listed below.

3.1.1 Antioxidant enzyme activity determination

For enzyme extraction, 200 mg of frozen leaf tissue was mechanically homogenized in 4 mL of chilled 50 mM potassium phosphate buffer (pH 7.0). The extraction buffer contained 2 mM sodium EDTA and 1% (w/v) polyvinylpyrrolidone (PVPP) to enhance protein stability and remove phenolic compounds. Following homogenization, the samples were centrifuged at 10,000 × g for 10 minutes at 4°C to pellet cellular debris. The clarified supernatants were carefully transferred to fresh micro-centrifuge tubes and immediately stored at -80°C to preserve enzyme activity until analysis. To ensure experimental reproducibility, all enzymatic assays were performed using independently prepared extracts from three separate biological replicates. Enzyme activity measurements were conducted within two weeks of sample preparation to minimize degradation effects.

3.1.2 Superoxide dismutase (SOD) activity

SOD activity was determined by measuring the inhibition of nitroblue tetrazolium (NBT) reduction in a photochemical reaction, adapted from Giannopolitis and Ries (1977). The 3 mL reaction mixture contained 50 mM potassium phosphate buffer (pH 7.8), 0.1 mM EDTA (pH 8.0), 14.9 mM methionine, 63 µM NBT, 90 µL of enzyme extract, and 8 µM riboflavin. The reaction was initiated by exposing the mixture to 4,000 lux light for 5 min, followed by immediate transfer to darkness to stop the reaction. Two control reactions were included: one kept in complete darkness and another exposed to light. The absorbance was recorded at 560 nm using a spectrophotometer. One unit (U) of SOD activity was defined as the enzyme quantity causing 50% inhibition of NBT reduction under the assay conditions.

3.1.3 Catalase (CAT) activity

CAT enzyme activity was measured spectrophotometrically by monitoring the decrease in absorbance at 240 nm resulting from H_2O_2 decomposition, according to the method described by Aebi (1984). The assay mixture contained 2.67 mL of 50 mM phosphate buffer (pH 7.0), to which 30 µL of enzyme extract and 300 µL of 100 mM H_2O_2 were added to initiate the reaction. The change in absorbance was recorded for 2 minutes at 25°C. CAT activity was determined using the molar extinction coefficient of 39.4 mM^{-1} cm^{-1} for H_2O_2, with one unit of enzyme activity defined as the amount required to decompose 1 µmol of H_2O_2 per minute under the assay conditions.

3.1.4 Peroxidase (POD) activity

POD activity was determined spectrophotometrically by measuring the oxidation of guaiacol at 470 nm, following the established protocol (Maehly and Chance, 1954). The 3 mL reaction system contained 2.84 mL of 10 mM potassium phosphate buffer (pH 7.0), 50 µL of 20 mM guaiacol solution, and 90 µL of enzyme extract. The enzymatic reaction was initiated by adding 20 µL of 40 mM H_2O_2. The increase in absorbance was monitored for 2 minutes at 25°C. POD activity was measured using the guaiacol extinction coefficient of 26.6 mM^{-1} cm^{-1}, with one enzyme unit defined as the amount required to produce 1 µmol of tetra-guaiacol per minute under the specified conditions.

3.1.5 Ascorbate peroxidase (APX) activity

The enzymatic activity of APX was evaluated by monitoring the oxidation rate of ascorbic acid at 290 nm wavelength, following the methodology established by Nakano and Asada (1981). The

assay system (1 mL total volume) contained the following components, including 50 mM potassium phosphate buffer (pH 7.0), 1 mM EDTA-Na_2 (as a stabilizing agent), 0.5 mM freshly prepared ascorbic acid (substrate), 0.1 mM hydrogen peroxide (H_2O_2, reaction initiator), and 25 µL of enzyme extract. The reaction was started by adding H_2O_2, and the decrease in absorbance was recorded for 2 minutes at 25°C. APX activity was calculated using the molar extinction coefficient of 2.8 mM^{-1} cm^{-1} for oxidized ascorbate. One unit of APX activity was defined as the amount of enzyme required to oxidize 1 µmol of ascorbate per minute under the specified assay conditions.

3.1.5 Proline content determination

The proline concentration in leaf tissues was measured using an optimized protocol based on the method by Bates et al. (1973). Leaf samples were collected 48 hours following stress treatment for analysis. The extraction procedure involved sample preparation (Fresh leaf tissue (200-300 mg) was homogenized in 2 mL of 3% (w/v) sulfosalicylic acid and the homogenate was centrifuged at 10,000 × g for 20 minutes at 4°C to remove cellular debris), colorimetric reaction (1 mL of supernatant was mixed with 1 mL of acid-ninhydrin reagent, containing 1.25 g ninhydrin dissolved in 30 mL glacial acetic acid and 20 mL 6 M phosphoric acid, and the mixture was heated at 100°C for 60 minutes in a water bath), proline extraction (The reaction was terminated by rapid cooling on ice, proline was extracted into 2 mL toluene by vigorous vortexing for 15 seconds, and the organic phase was separated after 60 minutes of dark incubation at 25°C), and spectrophotometric analysis (Absorbance of the toluene phase was measured at 520 nm using toluene as blank and proline concentration was determined from a standard curve [0-20 µg/Ml]). Results were expressed as micrograms per gram fresh weight ($\mu g \cdot g^{-1}$ FW) with standard error (n=3).

3.1.6 Malondialdehyde (MDA) content determination

The extent of lipid peroxidation in leaf tissues was determined through MDA measurement, following an adapted thiobarbituric acid (TBA) assay protocol (Heath and Packer, 1968). Samples were collected after 48 hours of stress treatment from the upper fully expanded leaves, with three biological replicates analyzed (two leaves per plant averaged for each replicate). The experimental procedure involved the sample preparation (Fresh leaf tissue [200-300 mg] was homogenized in 2 mL of 0.1% (w/v) TCA and the homogenate was centrifuged at 10,000 × g for 20 minutes at 4°C), TBA reaction (1.8 mL of supernatant was combined with 2 mL of TBA reagent [0.5% TBA in 20% TCA], the mixture was heated at 100°C for 30 minutes in a water bath, and Samples were immediately cooled on ice for 10 minutes to stop the reaction), spectrophotometric analysis (After centrifugation at 10,000 × g for 5 minutes, the supernatant was analyzed, absorbance readings were taken at 532 nm [primary peak] and 600 nm [background correction], and a reagent blank [0.5% TBA in 20% TCA] was used for baseline correction), and calculation (MDA concentration was determined using the following equation:

$$\text{MDA } (\mu mol \cdot g^{-1} \text{ FW}) = [(A_{532} - A_{600})/155{,}000] \times V \times (1/W)$$

where: 155,000 = molar extinction coefficient ($M^{-1}cm^{-1}$)

V = total volume (mL)

W = fresh tissue weight (g).
